# The Emergence of Successful Streptococcus pyogenes Lineages through Convergent Pathways of Capsule Loss and Recombination Directing High Toxin Expression

**DOI:** 10.1128/mBio.02521-19

**Published:** 2019-12-10

**Authors:** Claire E. Turner, Matthew T. G. Holden, Beth Blane, Carolyne Horner, Sharon J. Peacock, Shiranee Sriskandan

**Affiliations:** a Molecular Biology & Biotechnology, The Florey Institute, University of Sheffield, Sheffield, United Kingdom; b Department of Infectious Disease, Imperial College London, London, United Kingdom; c Pathogen Genomics, The Wellcome Trust Sanger Institute, Cambridge, United Kingdom; d School of Medicine, University of St Andrews, St Andrews, United Kingdom; e Department of Medicine, University of Cambridge, Cambridge, United Kingdom; f British Society for Antimicrobial Chemotherapy, Birmingham, United Kingdom; Northern Arizona University

**Keywords:** group A *Streptococcus*, NADase, *Streptococcus pyogenes*, streptolysin O, convergent evolution, homologous recombination, hyaluronic acid capsule, whole-genome sequencing

## Abstract

Gene transfer and homologous recombination in Streptococcus pyogenes has the potential to trigger the emergence of pandemic lineages, as exemplified by lineages of *emm*1 and *emm*89 that emerged in the 1980s and 2000s, respectively. Although near-identical replacement gene transfer events in the *nga* (NADase) and *slo* (streptolysin O) loci conferring high expression of these toxins underpinned the success of these lineages, extension to other *emm* genotype lineages is unreported. The emergent *emm*89 lineage was characterized by five regions of homologous recombination additional to *nga-slo*, including complete loss of the hyaluronic acid capsule synthesis locus *hasABC*, a genetic trait replicated in two other leading *emm* types and recapitulated by other *emm* types by inactivating mutations. We hypothesized that other leading genotypes may have undergone similar recombination events. We analyzed a longitudinal data set of genomes from 344 clinical invasive disease isolates representative of locations across England, dating from 2001 to 2011, and an international collection of S. pyogenes genomes representing 54 different genotypes and found frequent evidence of recombination events at the *nga*-*slo* locus predicted to confer higher toxin genotype. We identified multiple associations between recombination at this locus and inactivating mutations within *hasAB*, suggesting convergent evolutionary pathways in successful genotypes. This included common genotypes *emm*28 and *emm*87. The combination of no or low capsule and high expression of *nga* and *slo* may underpin the success of many emergent S. pyogenes lineages of different genotypes, triggering new pandemics, and could change the way S. pyogenes causes disease.

## Abstract (precis)

Streptococcus pyogenes is a genetically diverse pathogen, with over 200 different genotypes defined by *emm* typing, but only a minority of these genotypes are responsible for the majority of human infection in high-income countries. Two prevalent genotypes associated with disease rose to international dominance following recombination of a toxin locus that conferred increased expression. Here, we found that recombination of this locus and promoter has occurred in other diverse genotypes, events that may allow these genotypes to expand in the population. We identified an association between the loss of hyaluronic acid capsule synthesis and high toxin expression, which we propose may be associated with an adaptive advantage. As S. pyogenes pathogenesis depends both on capsule and toxin production, new variants with altered expression may result in abrupt changes in the molecular epidemiology of this pathogen in the human population over time.

## INTRODUCTION

The capacity for the bacterial human pathogen Streptococcus pyogenes to undergo genetic exchange, independent of known bacteriophages or mobile elements, is not well understood, yet recent evidence suggests it underpins the emergence of successful new variants that rapidly rise to international dominance. Homologous recombination of a chromosomal region encompassing the toxin genes *nga* (encoding NADase), *ifs* (encoding the inhibitor for NADase), and *slo* (encoding streptolysin O), which was dated to have occurred in the mid-1980s, is thought to have driven the rise of *emm*1 to almost global dominance (1). The homologous recombination event resulted in increased *nga*-*slo* expression compared to that of the previous variant, linked to the gain of a highly active *nga-ifs-slo* promoter in the new *emm*1 variant compared to that of the previous variant (2).

A very similar recombination event was recently identified in the genotype *emm*89. A new variant of *emm*89 sequence type (ST) 101 (also referred to as clade 3) emerged, having undergone six regions of predicted homologous recombination compared to its ST101 predecessor (also referred to as clade 2) (3, 4). One of the six regions encompassed the *nga-ifs-slo* locus, comprising a region almost identical to that of *emm*1, which conferred similarly high expression of *nga* and *slo* compared to that of the previous variant. Another recombination region within the emergent ST101 *emm*89 resulted in the loss of the hyaluronic acid capsule. We dated the emergence of this new acapsular high-toxin-expressing ST101 *emm*89 lineage to the mid-1990s, but there was a rapid increase and rise to dominance in the United Kingdom between 2005 and 2010 (3). The lineage is now the dominant form of *emm*89 in the United Kingdom as well as other parts of the world, including Europe, North America, and Japan (4–8).

Given that recombination associated with *nga*-*ifs*-*slo* can give rise to new successful S. pyogenes variants, we hypothesized that this may be a feature common to other successful *emm* types. To determine if this is the case, we sequenced the genomes of 344 S. pyogenes invasive disease isolates originating from hospitals across England between 2001 and 2011 and compared the data with other available historical and contemporary international S. pyogenes whole-genome sequence (WGS) data. We identified that recombination of the *nga-ifs-slo* locus has occurred in other leading *emm* types, supporting the hypothesis that it can underpin the emergence and success of new lineages. We also identified an association of *nga-ifs-slo* recombination toward a high-activity promoter variant with inactivating mutations within the capsule locus. This suggests that loss of capsule may also provide an advantage to certain genotypes, either through a direct effect on pathogenesis or an association with the process of recombination.

## RESULTS

### Genetic characterization of bacteremia isolates.

We performed whole-genome sequencing of 344 S. pyogenes invasive isolates collected from hospitals across England by the British Society for Antimicrobial Chemotherapy (BSAC) Bacteraemia Resistance Surveillance Program from 2001 to 2011. Forty-four different *emm* types were identified from *de novo* assembly, with the most common being *emm*1 (*n* = 64, 18.6%), *emm*12 (*n* = 34, 9.9%), *emm*89 (*n* = 32, 9.3%), *emm*3 (*n* = 28, 8.1%), *emm*87 (*n* = 22, 6.4%), and *emm*28 (*n* = 15, 4.4%) (see Fig. S1 in the supplemental material). Antimicrobial susceptibilities were typical for S. pyogenes, with 100% of isolates susceptible to penicillin and 22% resistant to clindamycin, erythromycin, and/or tetracycline; detailed susceptibilities and associated genotypes are reported in Data Set S1.

10.1128/mBio.02521-19.1 FIG S1 Number of isolates per *emm* type in the BSAC collection. Forty-four different genotypes were identified within the collection, but 16 were represented by single isolates (grey bars). Total number of isolates was 344. Download FIG S1, TIF file, 0.2 MB. Copyright © 2019 Turner et al. 2019 Turner et al. This content is distributed under the terms of the Creative Commons Attribution 4.0 International license.

10.1128/mBio.02521-19.8 DATA SET S1 Details of BSAC isolates and antimicrobial sensitivity testing. Download Data Set S1, XLSX file, 0.1 MB. Copyright © 2019 Turner et al. 2019 Turner et al. This content is distributed under the terms of the Creative Commons Attribution 4.0 International license.

**FIG 1:**
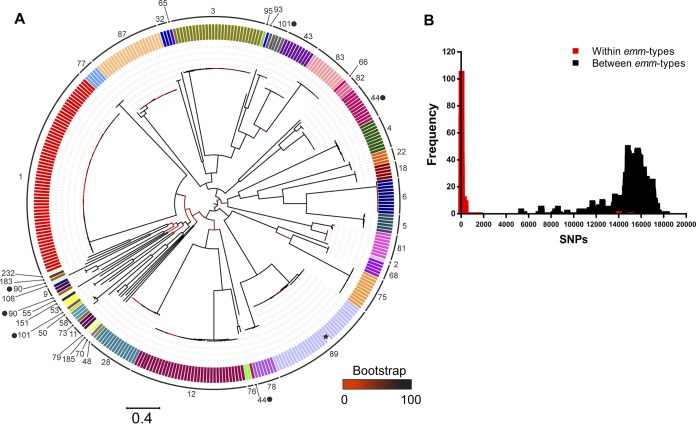
Low diversity within *emm* genotypes. (A) A maximum likelihood phylogenetic tree constructed from 113,805 core SNPs extracted after mapping all 344 BSAC isolates to the complete *emm*89 reference strain H293 (indicated by a star) identified that the majority of isolates cluster by *emm* genotype. Exceptions were *emm*44, *emm*90, and *emm*101 (highlighted with black dots), each of which were present as two separate lineages. Branches are colored based on bootstrap support (scale bar in figure). Boxes at branch tips are colored by *emm* type, and the *emm*-type numbers are provided outside the tree. (B) As reflected by the phylogenetic tree, the number of SNPs separating isolates was high (>5,000) when the genomes of isolates of different *emm* types were compared (black bars). This was much lower when comparisons were made between the genomes of isolates of the same *emm* type (red bars).

The phylogenetic distribution of the 344 isolates based on core genome variation revealed distinct clustering by *emm* type, each forming single lineages with the exceptions of *emm*44, *emm*90, and *emm*101, each of which formed two lineages (Fig. 1A). Pairwise distances between isolates gave a median of just 45 single nucleotide polymorphisms (SNPs) separating the genomes of isolates of the same *emm* genotype (range, 0 to 15,137 SNPs) compared to a median of 15,648 SNPs separating the genomes of isolates of different *emm* types (range, 5,312 to 18,317 SNPs) (Fig. 1B). The genotypes *emm*44, *emm*90, and *emm*101 gave the highest SNP distance for the intra-*emm* comparison (13,494 to 15,137 SNPs), which approaches the median level observed between *emm* types. This indicated that while other genotypes represent a relatively conserved chromosomal genetic background, the populations of *emm*44, *emm*90, and *emm*101 exhibit more diverse chromosomal backgrounds despite representing the same *emm* type, potentially due to *emm* gene switching.

### High level of variation within the *nga-ifs-slo* locus.

**FIG 2:**
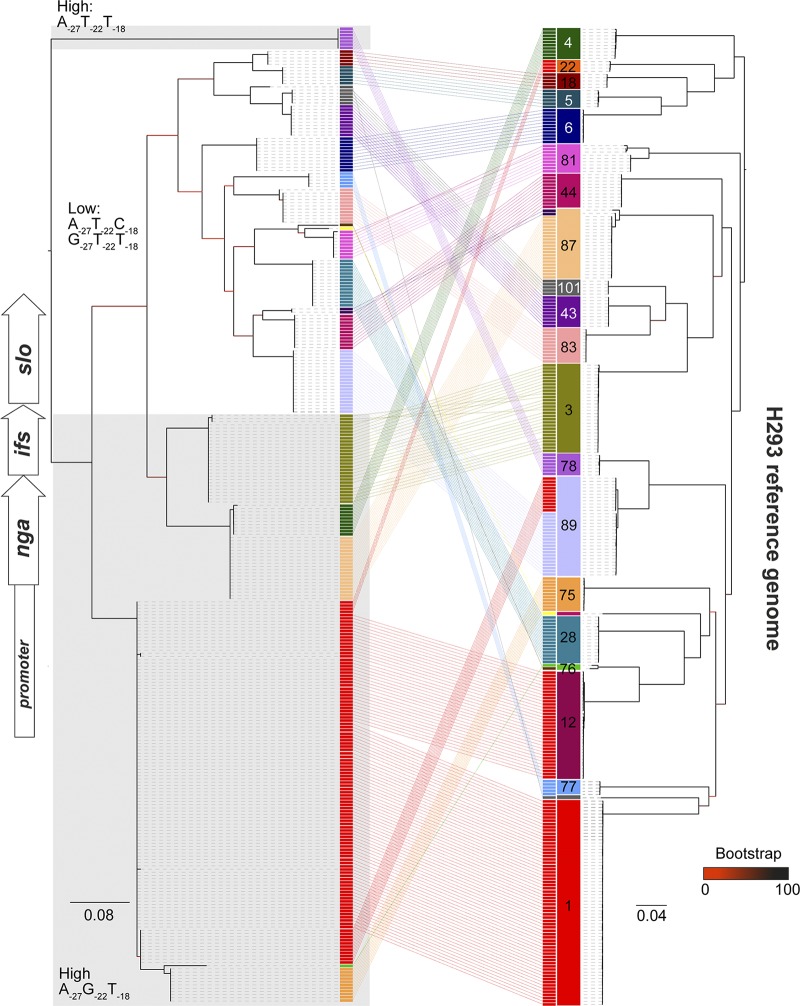
Comparison of the variation within the P-*nga-ifs-slo* region and core chromosome. A maximum likelihood phylogenetic tree was constructed from 205 SNPs extracted from an alignment of the *nga-ifs-slo* locus and associated upstream region to include the promoter (P-*nga-ifs-slo*) extracted from *de novo* assemblies of BSAC S. pyogenes collection (left tree). This was compared to the phylogenetic tree constructed using 75,851 SNPs across the entire core genome after mapping to the H293 reference genome (right tree). Only 20 of the most common *emm* genotypes were included: *emm*1, -3, -4, -5, -6, -12, -18, -22, -28, -43, -44, -75, -76, -77, -78, -81, -83, -87, -89, and -101 (*n* = 303 isolate genomes). Numbers and colored blocks on the right tree represent *emm* types. Variants of the P-*nga-ifs*-*slo* are of the same color as the *emm* type if unique to that *emm* type. The P-*nga-ifs-slo* variant found in *emm*1 (red) was common to other genotypes of *emm*12, *emm*22, and some *emm*89 isolates. The genotypes *emm*76, *emm*87, and *emm*89 were linked to more than one variant of P-*nga-ifs-slo*. Gray shading indicates high-expressing promoter variants: A_−27_T_−22_T_−18_ (top) or A_−27_G_−22_T_−18_ (bottom). Other nonshaded areas are low-expressing promoter variants A_−27_T_−22_C_−18_ or G_−27_T_−22_T_−18._ Scale bar represents substitutions per site. Bootstrap support values are provided on branches.

To identify the level of variation within the *nga-ifs-slo* locus, we extracted the sequence from the 3′ end of *nusG* (immediately upstream of *nga*) to the 3′ end of *slo* (P-*nga-ifs-slo*), comprising the entire locus and all of the upstream sequence, including the predicted ∼67-bp *nga-ifs-slo* promoter region (9). We constructed a phylogenetic tree from SNPs within the P-*nga-ifs-slo* region from the genomes of isolates belonging to the most common *emm* types and compared it to the phylogeny constructed with SNPs extracted from a whole-genome comparison to a reference *emm*89 genome, H293 (Fig. 2). Most *emm* genotypes were associated with a single P-*nga-ifs-slo* variant that was unique to that genotype. The main exception to this was the P-*nga-ifs-slo* variant found in modern (post-1980s M1T1) *emm*1, as this was also found in all *emm*12, all *emm*22 (a lineage known to be acapsular) isolates, and 11 of the 32 *emm*89 isolates. These 11 *emm*89 isolates represented the emergent acapsular ST101 variant, while the remaining 21 *emm*89 isolates represented the original encapsulated ST101 variant, with a different unique P-*nga-ifs-slo* as previously reported (3). The entire *emm*75 population and one of the two *emm*76 isolates were also associated with a P-*nga-ifs-slo* variant that was closely related to the *emm*1-like variant. All but two *emm*87 isolates had a P-*nga-ifs-slo* variant also found in the acapsular lineage *emm*4. The presence of multiple P-*nga-ifs-slo* variants within the *emm*76 and *emm*87 genotypes, where the core chromosome was otherwise relatively conserved, indicated that gene transfer and recombination are responsible for the P-*nga-ifs-slo* variation in these genotypes rather than extensive genome-wide divergence or *emm* “switching.”

### Variants of the *nga-ifs-slo* promoter associated with altered expression.

**FIG 3:**
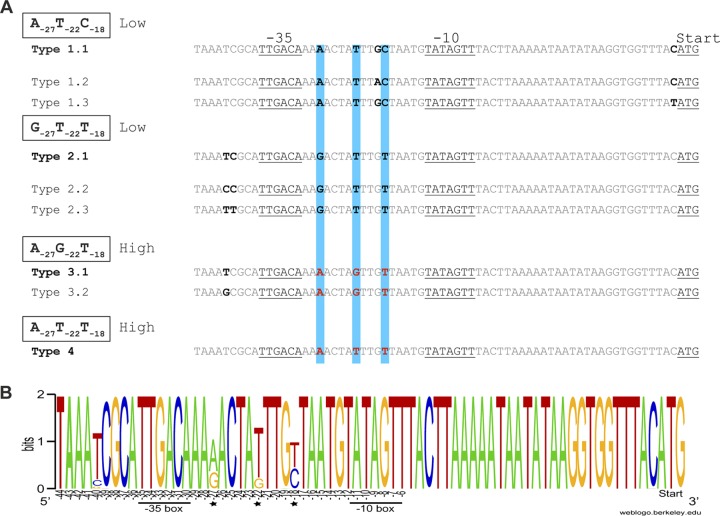
Variants of the *nga-ifs-slo* promoter. (A) The three key residues predicted to influence promoter activity are highlighted in blue, with those associated with high activity in red font. We identified four combinations of these residues (four promoter types) with subtype variants differing in residues other than −27, −22, and −18 (residue positions relative to the underlined −35 and −10 regions) in the predicted 67-bp promoter region (9). The combination of A_−27_T_−22_C_−18_ subtype 1.1 in historical *emm*1 and G_−27_T_−22_T_−18_ subtype 2.1 in older *emm*89 isolates has been shown to be associated with low-level promoter activity. A_−27_G_−22_T_−18_ subtype 3.1 promoter in modern *emm*1 and emergent variant *emm*89 has been shown to have high activity. A_−27_T_−22_T_−18_ subtype 4 promoter has also been shown to have high activity in *emm*28 (15). Subtypes 1.2, 1.3, and 2.3 were restricted to *emm*9, *emm*88, and *emm*32 strains, respectively. (B) WebLogo representation of the variability in the 67-bp promoter region of *nga-ifs-slo* within the 54 different *emm* types. Key residues −27, −22, and −18 are highlighted (star) and their positions are relative to the −35 and −10 boxes. Figure generated using WebLogo (http://weblogo.berkeley.edu).

Recombination of P-*nga-ifs-slo* and surrounding regions in *emm*1 and *emm*89 conferred higher activity and expression of NGA (NADase) and SLO (1, 3, 10). This change in expression was linked to the combination of three key residues at −27, −22, and −18 within the *nga-ifs-slo* promoter. A_−27_G_−22_T_−18_ at these key sites was associated with high *nga-ifs-slo* promoter activity in *emm*1 and emergent *emm*89 following recombination (also referred to as Pnga3) compared to low promoter activity of historical *emm*1 and *emm*89, associated with the key site combinations A_−27_T_−22_C_−18_ and G_−27_T_−22_T_−18_, respectively (2) (Fig. 3A). We compared the ∼67-bp *nga-ifs-slo* promoter region of the 344 BSAC collection isolate genomes to identify different variants. We expanded the data analyzed by including assembled genome data from >5,000 isolates representing 54 different *emm* types: from Cambridge University Hospital (CUH) (11), from England and Wales collected by Public Health England (PHE) in 2014 and 2015 (PHE-2014/15) (12, 13), and from the United States collected by the Active Bacterial Core Surveillance System (ABCs) in 2015 (ABCs-2015) (14). We excluded 39 *emm* types represented by fewer than 3 isolates (Data Set S2).

10.1128/mBio.02521-19.9 DATA SET S2 Details of all isolates with assembly statistics, capsule gene mutations, and *nga-ifs-slo* promoter variants. Download Data Set S2, XLSX file, 0.6 MB. Copyright © 2019 Turner et al. 2019 Turner et al. This content is distributed under the terms of the Creative Commons Attribution 4.0 International license.

Four combinations of the −27, −22, and −18 residues were found across all 5,271 isolates (Table 1); variant 1 A_−27_T_−22_C_−18_ and variant 2 G_−27_T_−22_T_−18_ are associated with low promoter activity, while variant 3 A_−27_G_−22_T_−18_ and variant 4 A_−27_T_−22_T_−18_ are associated with high promoter activity. We also identified subtypes of the 67-bp promoter region which varied at bases other than −27, −22, and −18 (Fig. 3A and B; Table 1). A_−27_T_−22_C_−18_ variant subtype 1.1 and G_−27_T_−22_T_−18_ variant subtype 2.1 were both previously confirmed to have low promoter activity (2) and were the most common variants found across genotypes. Other subtypes of these variants were restricted to single genotypes except G_−27_T_−22_T_−18_ variant subtype 2.2, which differed by a single substitution of C for a T residue at −40 bp. Two subtypes of the high-activity variant A_−27_G_−22_T_−18_ were found, the most common being subtype 3.1, associated with *emm*1 and emergent *emm*89, and subtype 3.2, which was found predominantly in the genomes of *emm*4 and *emm*87 and differed from subtype 3.1 by a single substitution of G for T at −40 bp. We measured the activity of NADase in the culture supernatants of strains representing different promoter subtypes and found that the presence of T/G/C at −40 bp did not affect activity of the promoter (see Fig. S2). The fourth promoter variant, A_−27_T_−22_T_−18_, is also associated with high activity (15) and was identified in the genomes of *emm*28, *emm*75, and all *emm*78 isolates. Only three *emm* types were exclusively associated with the high-activity promoter variant A_−27_G_−22_T_−18_: *emm*1, *emm*3, and *emm*12. Other *emm* types with the high-activity promoter variant also had one or more of the other three promoter variants, suggesting a mixed population or, as in the case of *emm*89, an evolving population.

**TABLE 1:** Three key residue variants within the *nga-ifs-slo* promoter

Promoter variant	Type	Genotype (% of isolates)^*a*^
A_−27_T_−22_C_−18_	1.1	**4 (1)**, 8 (100), **9 (92)**, 11 (100), **22 (3)**, **25 (33)**, **28 (87.7)**, 33 (100), 41 (100), 43 (100), **44 (9)**, 49 (100), 53 (100), **58 (15)**, 60 (100), 63 (100), **75 (9)**, **76 (41)**, **77 (29)**, **81 (23)**, **82 (1)**, **88 (33)**, **89 (1)**, **90 (4)**, 92 (100), **94 (6)**, 101 (100), **102 (50)**, **103 (17)**, 106 (100), **108 (89)**, 110 (100), 113 (100), 151 (100), 168 (100), 232 (100)
	1.2	9 (8)
	1.3	88 (67)
G_−27_T_−22_T_−18_	2.1	5 (100), 6 (100), 18 (100), **25 (67)**, **44 (28)**, 68 (100), **75 (1)**, **76 (5), 77 (1), 82 (1), 87 (2), 89 (6), 90 (96),** 91 (100)**, 102 (50), 103 (83),** 104 (100), 118 (100)
	2.2	2 (100), 27 (100), **44 (62)**, **58 (85)**, 59 (100), 73 (100), **76 (11)**, **77 (36)**, **82 (89)**, 83 (100)
	2.3	32 (100)
A_−27_G_−22_T_−18_	3.1	1 (100), 3 (100), 12 (100), **22 (97)**, **75 (90)**, **76 (43)**, **81 (77)**, **82 (9)**, **89 (93)**, **94 (94)**, **108 (11)**
	3.2	**4 (99), 28 (0.3), 77 (34)**, **87 (98)**
A_−27_T_−22_T_−18_	4	**28 (12)**, 78 (100)

a Boldface font indicates genotypes with more than one variant within the population.

10.1128/mBio.02521-19.2 FIG S2 NADase activity of different promoter subtypes. The activity of NADase was measured in culture supernatants of BSAC isolates representing different promoter subtypes with predicted low (black) or high (red) activity. A_−27_T_−22_C_−18_ subtype 1.1 promoter had low activity in *emm*81 isolates, consistent with previous findings of this promoter in historical *emm*1. G_−27_T_−22_T_−18_ subtype 2.1 had low activity in older *emm*89 isolates, also consistent with previous findings, and subtype 2.2 in *emm*58 and *emm*77 also had low activity, as predicted despite the additional base change at −40 bp. Compared to G_−27_T_−22_T_−18_ subtype 2.1 (older *emm*89), significantly higher activity was detected in *emm*1, with A_−27_G_−22_T_−18_ subtype 3.1, and in *emm*4 and *emm*87, with subtype 3.2, also supporting a null effect of the base change at −40 bp. A_−27_T_−22_T_−18_ subtype 4 promoter in *emm*78 also had significantly higher activity. Isolates with mutations in regulators *covR*S or *rocA* were excluded as they influence the expression of *nga*. Data represent means + standard deviations (SDs) for e*mm*1, *n* = 10; *emm*89, *n* = 11; *emm*58, *n* = 3; *emm*77, *n* = 3; *emm*4, *n* = 7; *emm*87, *n* = 17; *emm*78, *n* = 5; and *emm*81, *n* = 5. Statistical comparisons were made to *emm*89 subtype 2.1, for which we had the highest number of representative isolates and was previously confirmed to have low activity, using Kruskal-Wallis nonparametric multiple-comparison test. *, *P* < 0.05; **, *P* < 0.01; ***, *P* < 0.001; N.S., not significant. Download FIG S2, TIF file, 0.1 MB. Copyright © 2019 Turner et al. 2019 Turner et al. This content is distributed under the terms of the Creative Commons Attribution 4.0 International license.

We sought evidence for acquisition of the high-activity-associated promoter A_−27_G_−22_T_−18_ variant by *emm* genotypes where the dominant or ancestral state was a low-activity-associated promoter; these included (in addition to the aforementioned *emm*89) *emm*75, *emm*76, *emm*77, *emm*81, *emm*82, *emm*87, *emm*94, and *emm*108, all of which are *emm* types frequently identified in the United Kingdom and the United States (12–14). Although one *emm*28 was found to carry the high-activity-associated A_−27_G_−22_T_−18_ promoter, the rest of the *emm*28 population was divided between either A_−27_T_−22_C_−18_ or A_−27_T_−22_T_−18_ variants. The data pointed to a switch in P-*nga-ifs-slo* in all cases rather than an *emm* switch, except for *emm*82, where the *emm*82 gene replaced the *emm*12 gene in an *emm*12 genetic background (14).

### High level of mutations within the capsule locus leading to truncations of HasA or HasB.

**FIG 4:**
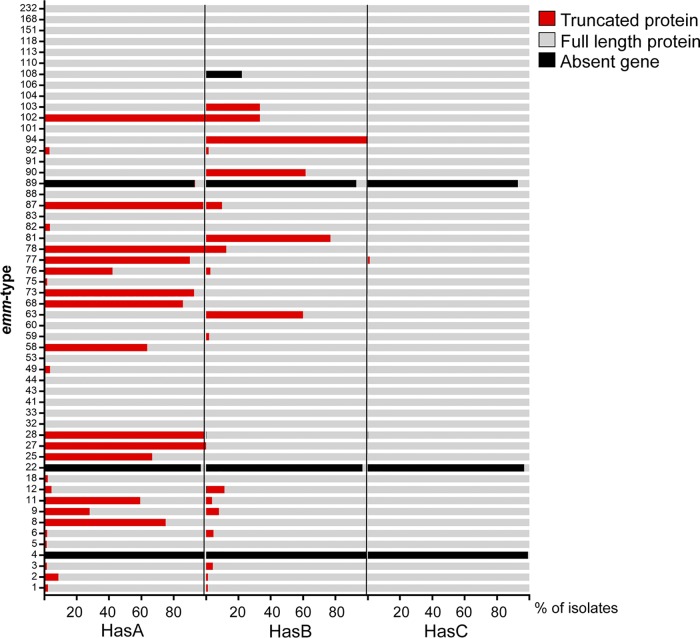
Nonfunctional mutations within the capsule locus genes. The *hasABC* genes were extracted from the assembled genomes of BSAC, CUH, PHE-2015/15, and ABCs-2015 isolate genome collections, and polymorphisms or indels leading to nonsense mutations and premature stop codons, as well as gene absence, were identified. The percentage of isolates with full-length (gray), truncated (red), or absent (black) HasA, HasB, or HasC is depicted for each of the 54 *emm* types. *emm* types with fewer than 3 isolates were excluded. *N* = 5,271 isolates genomes shown. Mutations in HasA were detected in more than 50% of isolates belonging to genotypes *emm*8 (*n* = 3/4), *emm*11 (*n* = 63/108), *emm*25 (*n* = 2/3), *emm*27 (*n* = 3/3), *emm*28 (*n* = 358/363), *emm*58 (*n* = 21/33), *emm*68 (*n* = 12/14), *emm*73 (*n* = 25/27), *emm*77 (*n* = 72/80), *emm*78 (*n* = 8/8), *emm*87 (*n* = 119/121), and *emm*102 (*n* = 6/6). Mutations in HasB were detected in 100% of *emm*94 isolates (*n* = 54/54) and 60% to 77% of *emm*63 (*n* = 3/5), *emm*81 (*n* = 50/65), and *emm*90 (*n* = 16/26) isolates.

As well as recombination around the P-*nga-ifs-slo* region, the emergent ST101 variant of *emm*89 had also undergone recombination surrounding the *hasABC* locus, and, in place of the *hasABC* genes, there was a region of 156 bp that was not found in genotypes with the capsule locus but is found in the acapsular *emm*4 and *emm*22 isolates (3). To identify any similar events in other genotypes, we examined the sequences of *hasA*, *hasB*, and *hasC* in the assemblies of isolates from the BSAC collection as well as CUH (11), PHE-2014/15 (12, 13), and ABCs-2015 (14) collections for gene presence as well as premature stop codon mutations (Fig. 4). The *hasABC* locus was absent in the majority of *emm*89 isolates, consistent with the previous observations describing the recent emergence of the acapsular *emm*89 variant (3). Similarly, the *hasABC* genes were absent in all *emm*4 and *emm*22 isolates, as previously identified (16), except for two *emm*4 isolates and one *emm*22 isolate which had an intact *hasABC* locus predicted to encode full-length proteins. We confirmed the genotypes of these isolates by *emm* typing the assembled genomes. Multilocus sequence typing (MLST) and phylogenetic analysis indicated that they both had a very different genetic background to other *emm*4 or *emm*22 populations, suggesting that these were not typical of these *emm* types; therefore, they represent examples of *emm* switching. Interestingly, we also identified a similar replacement of *hasABC* for the 156-bp region in one *emm*28 isolate (PHE-2014/15, GASEMM1261 [13]), but phylogenetic analysis suggested this was highly divergent from the rest of the *emm*28 population, likely to represent another example of *emm* switching. Isolated examples of individual *hasA* or *hasB* gene loss were identified in the genomes of isolates belonging to *emm*1 (*n* = 1), *emm*3 (*n* = 1), *emm*11 (*n* = 1), *emm*12 (*n* = 4), and *emm*108 (*n* = 2).

The majority of genotypes (35/54 [65%]) had isolates without genes or truncation mutations in at least one of the *hasABC* genes (Fig. 4). Mutations in *hasC* were rare and only detected in one isolate, an *emm*77 isolate, which also had a mutation within *hasA*. Within seven of the eight *emm* types for which we identified potential P-*nga-ifs-slo* recombination, a high percentage of isolates had inactivating mutations in *hasA* and *hasB*, suggesting a possible association between an acapsular genotype/phenotype and recombination of P-*nga-ifs-slo* to gain a high-activity promoter. Including the previously identified *emm*1 and *emm*89 recombination events, P-*nga-ifs-slo* recombination to gain a high-activity promoter was detected in 10 genotypes, and in all 10 of these genotypes (100%) were isolates with *hasAB* gene mutations or gene absence. However, in the 44 genotypes that had not undergone P-*nga-ifs-slo* recombination to gain a high-activity promoter, significantly fewer (25/44 [57%]) had isolates with a *hasAB* gene mutation or gene absence (χ^2^_1df_ = 6.662, *P* = 0.0098).

### Recombination of P-*nga-ifs-slo* and surrounding regions.

**FIG 5:**
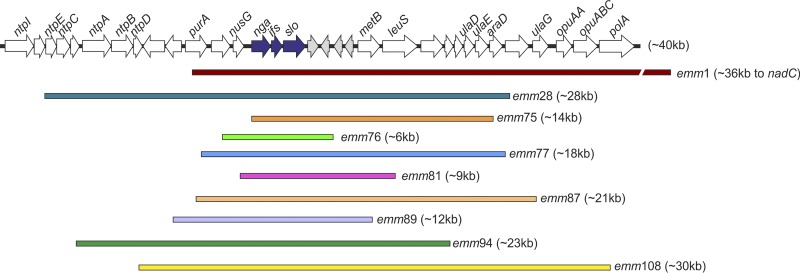
Regions of recombination spanning the P-*nga-ifs-slo* locus. Recombination across the *nga*, *ifs*, and *slo* genes (blue arrows) was identified in eight genotypes in addition to the previously described *emm*1 and *emm*89. Length of recombination, predicted by SNP cluster analysis, ranged from ∼6 kb to 36 kb. With the exception of *emm*75, all regions also encompassed the promoter of *nga-ifs-slo*. All regions are shown relative to an ∼40-kb region within the reference genome H293, and genes within this region are depicted as arrows. Recombination in *emm*1 extended beyond that depicted here and is shown as a broken line.

To confirm our prediction that genotypes *emm*28, *emm*75, *emm*76, *emm*77, *emm*81, *emm*87, *emm*94, and *emm*108 had undergone recombination around P-*nga-ifs-slo*, we mapped all the genome sequence data for each genotype to the *emm*89 reference genome H293. Gubbins analysis of SNP clustering predicted regions of recombination spanning the *nga*-*ifs-slo* region and varied in length in all eight genotypes (Fig. 5). To further analyze the recombination of these genotypes and potential capsule loss, we studied the population structure of each genotype individually.

### Recombination within *emm*28 and *emm*87 around P-*nga-ifs-slo* and the capsule locus.

The genotypes *emm*28 and *emm*87 were the sixth and fifth most common in the BSAC collection, respectively, and *emm*28 was previously noted to be a major cause of infection in high-income countries (17). We focused attention on *emm*28 and *emm*87, as there has been little genomic work on these genotypes so far.

**FIG 6:**
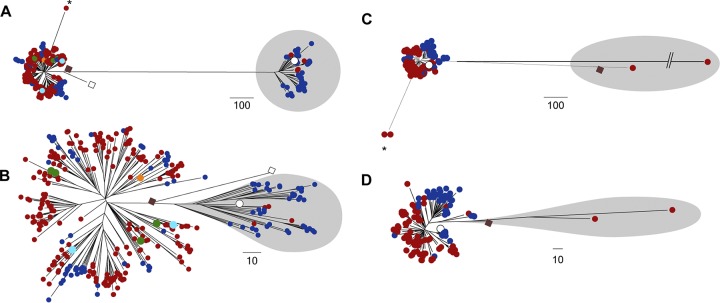
Recombination within the *emm*28 and *emm*87 populations. (A) Maximum likelihood phylogeny constructed with 33,537 core SNPs following mapping of all available *emm*28 genome data to the *emm*28 MGAS6180 reference genome (white square) (18). Modern UK isolates (red circles): BSAC (*n* = 15), CUH (*n* = 13 [11]), and PHE-2014/15 (*n* = 240 [12, 13]); one historical English isolate from 1938 (brown square). North American isolates (blue circles): ABCs-2015 (*n* = 95 [14]), Canada (2011 to 2013, *n* = 4 [47]), and completed genome strain HarveyGAS (United States, 2017 [48]). Other isolates: Lebanon (*n* = 1, orange circle [49]), Australia (*n* = 5, green circles [50]), and France (STAB10015 [51], M28PF1 [52], turquoise circles). Total number of isolate genomes was 379. Two lineages of *emm*28 were identified, one clustering with MGAS6180 (white square) and the other (shaded gray) clustering with MEW123 (2012 USA [19], white circle). (B) Regions of recombination were then identified within the *emm*28 genome alignment and removed before reconstructing a phylogenetic tree using 17,885 variable sites. (C) Maximum likelihood phylogeny constructed with 6,292 core SNPs following mapping of all available *emm*87 genome sequence data to the reference *emm*87 strain NGAS743 (Canada, white circle [53]). UK isolates (red circles): BSAC (2001 to 2011, *n* = 22), CUH (2008, *n* = 1 [11]), and PHE-2014/15 (*n* = 72, [12, 13]). North American isolates (blue circles): ABCs-2015 (*n* = 26 [14]), Canada (*n* = 23 [47, 53]), and Texas Children’s Hospital (2012 to 2016, *n* = 27 [54]). NCTC12065 (GenBank accession number GCA_900460075.1) isolate from ∼1970 to 1980 was also included (brown square). Total number of isolates was 173. Three isolates (shaded gray) were distinct from the main population. The branch was shortened for one isolate for presentation purposes. (D) Regions of recombination were identified within the *emm*87 genome alignment and removed before reconstructing a phylogenetic tree using 1,531 variable sites. Isolates indicated by an asterisk (*) in both *emm*28 and *emm*87 populations were predicted to have undergone recombination in regions surrounding the *hasABC* locus. Scale bars represent single nucleotide polymorphisms. PHE-2014/15 *emm*28 isolates GASEMM1261, GASEMM2648, GASEMM1396, and GASEMM1353 were removed for presentation purposes, as they represented highly divergent lineages.

All BSAC *emm*28 isolates carried the A_−27_T_−22_C_−18_ low-activity-associated promoter, but inclusion of international genomic data identified A_−27_T_−22_T_−18_ variant-carrying isolates. These two promoter variants were associated with different major lineages within the entire population of 379 international *emm*28 isolates, including one newly sequenced English isolate originally isolated in 1938. The majority of isolates (*n* = 373) clustered either with the reference MGAS6180 strain (United States) (18) or with the reference MEW123 strain (United States) (19) (Fig. 6A). Gubbins analysis for core SNP clustering predicted that the two lineages were distinguished by a single 28,200-bp region of recombination, between positions 142,426 bp (*ntpE*; M28_Spy0126) and 170,625 bp (M28_Spy0153) of the MGAS6180 chromosome. This suggests the emergence of one lineage from the other through a single recombination event followed by expansion of both lineages (Fig. 6B). Within the recombination region was the P-*nga-ifs-slo* locus, which differed between the two lineages; although unique in the MGAS6180-like lineage and with low-activity-associated promoter residues A_−27_T_−22_C_−18_, the MEW123-like lineage had a P-*nga-ifs-slo* identical to that found in *emm*78 isolates, with the three key residues of A_−27_T_−22_T_−18_. This is supported by recent findings identifying two main lineages within *emm*28 and that the A_−27_T_−22_T_−18_ promoter variant conferred greater toxin expression than A_−27_T_−22_C_−18_ (15).

Although we identified an A_−27_G_−22_T_−18_ high-activity variant of P-*nga-ifs-slo* within *emm*28, this was only associated with the highly divergent GASEMM1261 isolate that may represent an *emm* switching event. This isolate, along with three other PHE-2014/15 isolates (GASEMM2648, GASEMM1396, and GASEMM1353), also representing highly divergent lineages, were excluded from the phylogenetic analysis.

All *emm*28 isolates, regardless of lineage and including MGAS6180 (originally isolated in the 1990s), had the same insertion mutation within *hasA* of an A residue after 219 bp. This insertion was predicted to lead to a frameshift and a premature stop codon after 72 amino acids (aa) instead of full-length 420 aa, rendering *hasA* a pseudogene. Some isolates also had additional mutations in *hasA*: a deletion of an A residue in a septa-A tract leading to a frameshift and a stop codon after 7 aa (*n* = 1); a deletion of a T residue in a septa-T tract leading to a frameshift and a stop codon after 15 aa (*n* = 2); an insertion of an A residue after 57 bp leading to a frameshift and a stop codon after 46 aa (*n* = 3). The loss of full-length HasA would render the isolates acapsular.

In *emm*28, there were just two exceptions where *hasA* was found to be intact: the historical *emm*28 isolate from 1938 had an intact *hasABC* capsule operon, and BSAC_bs2099, which appeared to have undergone recombination to acquire a 22,316-bp region surrounding the *hasABC* genes that was 99% identical to the same region in *emm*2 isolate MGAS10270, suggesting *emm*2 might be the donor for this recombination. Both isolates were predicted to express full-length HasA and synthesize capsule. Taken together, in comparison with the oldest *emm*28 isolate, the data showed that post-1930s *emm*28 isolates became acapsular through mutation, but the contemporary population is divided into two major lineages, MEW123-like and MGAS6180-like lineages, that may differ in *nga-ifs-slo* expression. Additionally, there was evidence of geographical structure in the population: the MEW123-like lineage comprised mainly of North American isolates (39/44) and only five from England/Wales; isolates from Australia, France, and Lebanon were MGAS6180-like, along with the rest of the England/Wales isolates.

Phylogenetic analysis of the BSAC *emm*87 population was expanded and compared with publicly available *emm*87 genome sequence data, totaling 173 isolate genomes from the United Kingdom and North America, including one historical NCTC UK isolate from ∼1970 to 1980 (NCTC12065, GenBank accession number GCA_900460075.1). Gubbins analysis predicted a single 20,506-bp region of recombination surrounding the P-*nga-ifs-slo* region that distinguished the main population from the oldest BSAC isolates from 2001 and the historical 1970 to 1980 NCTC isolate (Fig. 6C). While the two 2001 BSAC isolates and the NCTC isolate had a P-*nga-ifs-slo* variant with low-activity-associated promoter residues, G_27_T_−22_T_−18_, all other *emm*87 isolates had a P-*nga-ifs-slo* region with high-activity-associated promoter residues, A_−27_G_−22_T_−18_, identical to that found in *emm*4 and some *emm*77 isolates. This suggested the emergence of a new lineage through a single recombination event followed by expansion within the population, redolent of that previously observed in *emm*89 (Fig. 6D).

Similar to *emm*28, all *emm*87 isolates, bar four, had an insertion of an A residue after 57 bp that resulted in a frameshift mutation in *hasA* and the introduction of a premature stop codon after 46 aa of HasA. This mutation was also identified within the historical NCTC isolate but was not found in the two 2001 BSAC isolates that had an intact *hasABC* locus. This mutation was also absent in two PHE-2014/15 isolates that had undergone an additional recombination event (32,243 bp) surrounding the *hasABC* locus; although, as this region shared 100% DNA identity to *emm*28 isolate MGAS6180, HasA is truncated. Overall the data showed that, like *emm*89 isolates, contemporary *emm*87 isolates are acapsular with a high-activity *nga-ifs-slo* promoter, suggesting that this *emm* lineage may have recently shifted toward this genotype/phenotype.

### Recombination within different multilocus sequence types of *emm*75.

**FIG 7:**
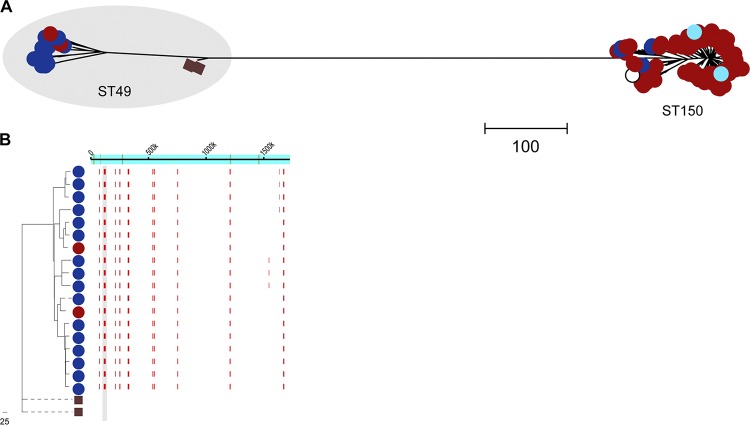
Two lineages within *emm*75. (A) Maximum likelihood phylogeny constructed with 9,241 core SNPs following mapping of all available *emm*75 genome sequence data to the genome of French strain STAB090229 (white circle) (55). Modern UK collections (red circles): BSAC (*n* = 11), CUH (*n* = 6 [11]), and PHE-2014/15 (*n* = 141 [12, 13]; two English historical isolates (brown squares) from 1937 and 1938. North American isolates (blue circles): ABCs-2015 (*n* = 20 [14]), NGAS344, and NGAS604 from Canada 2011 to 2012 (53). French strains (turquoise circles): STAB120304 (2012) and STAB14018 (2014) (55). Total number of isolates was 185. Two lineages were identified, generally characterized by the MLSTs: ST49 (shaded gray) or ST150 (with minor MSLT variants ST788, ST851, and ST861 within these lineages). (B) Gubbins analysis identified ten regions of predicted recombination (red lines) in all modern ST49 compared to historical 1930s ST49 across the genome (indicated across the top). One region included P-*nga-ifs-slo* (shaded gray). The phylogenetic tree was constructed with 1,953 variable sites following removal of predicted regions of recombination. Scale bars represent single nucleotide polymorphisms. One PHE-2014/15 isolate (GASEMM1722) was excluded for presentation purposes, as it was highly divergent from the rest of the population.

The *emm*75 genotype is of interest as a common cause of noninvasive infection in the United Kingdom; it is also used in models of nasopharyngeal infection (20, 21). Eleven *emm*75 isolates were present in the BSAC collection, all multilocus sequence type (ST) 150. When we incorporated other available genome sequence data for *emm*75 (*n* = 174), including two newly sequenced historical English *emm*75 isolates from 1937 and 1938, two major lineages were identified, characterized by two different MLSTs: ST49 or ST150 (Fig. 7A). Although the two historic English isolates were ST49, like the majority of modern North American isolates, the modern England/Wales isolates were predominantly ST150.

Although these two ST lineages differed in the P-*nga-ifs-slo* region, there was a high level of predicted recombination across the genomes of both STs, perhaps indicative of historic *emm* switching or extensive genetic exchange. ST49 isolates had the subtype 1.1 low-activity A_−27_T_−22_C_−18_ promoter, whereas all ST150 isolates had the A_−27_G_−22_T_−18_ subtype 3.1 high-activity promoter variant, identical to that of *emm*1 and *emm*89. Modern ST49 isolates did, however, differ from historic 1930s isolates by ten distinct regions of predicted recombination (Fig. 7B), including a region spanning the *nga-ifs-slo* locus, although this did not include the promoter region. We did not detect any mutations affecting the capsule region in *emm*75. Taken together, *emm*75 was characterized by two major MLST lineages differing in P-*nga-ifs-slo* promoter activity genotypes but without evidence of recent recombination or loss of capsule.

### Lineages associated with recombination in *emm*76, *emm*77, and *emm*81.

**FIG 8:**
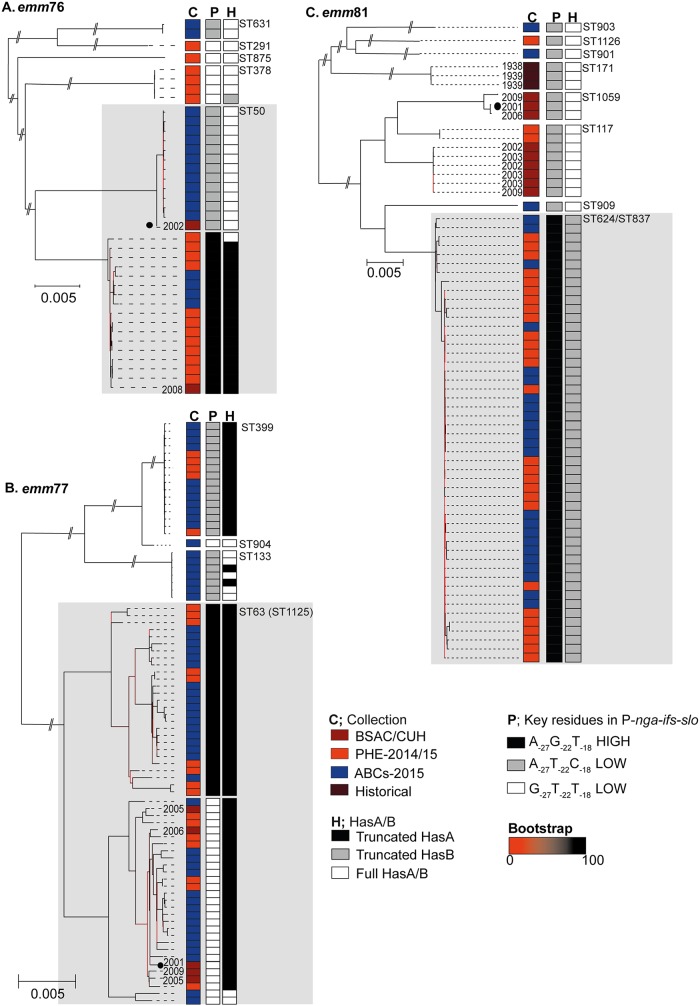
Variants of P-*nga-ifs-slo* and capsule mutations associated with lineages of *emm*76, *emm*77, and *emm*81. Maximum likelihood phylogeny identified multiple sequence type (ST) lineages within the populations of *emm*76 (A), *emm*77 (B), and *emm*81 (C). Collection indicates either BSAC or CUH (dark red), PHE-2014/15 isolates (red), ABCs-2015 (blue), or English historical (brown). Dates for BSAC, CUH, or historical are shown; other isolates were from 2014/2015. STs are indicated on the right and major lineages are shaded gray. (A) Genome data for *emm*76 was mapped to the *de novo* assembled sequence of BSAC_bs448 from 2002, selected as the oldest isolate representing the genotype. Genome data from a total of 38 isolates were used: BSAC (*n* = 2), PHE-2014/15 (*n* = 18 [12, 13]), ABCs-2015 (*n* = 18 [14]). Predicted prophage regions were removed and a maximum likelihood phylogenetic tree constructed from 30,264 core SNPs. Five STs were identified (indicated on right of tree), but the main lineage was ST50. (B) All *emm*77 genome data were mapped to the *de novo* assembled sequence of BSAC_bs150 from 2001. Genome data from a total of 80 isolates were used: BSAC (*n* = 5), PHE-2014/15 (*n* = 21 [12, 13]), and ABCs-2015 (*n* = 54 [14]). Four STs were identified but the main lineage was ST63, with one isolate in this lineage being single locus variant ST1125. Predicted prophage regions were removed, and a maximum likelihood phylogenetic tree was constructed from 34,760 core SNPs. (C) All *emm*81 genome data were mapped to the *de novo* assembled seqeunce of BSAC_bs229 from 2001. Genome data from a total of 68 isolates were used: BSAC (*n* = 9), CUH (*n* = 1 [11]), PHE-2014/15 (*n* = 29 [12, 13]), ABCs-2015 (*n* = 26 [14]), and English historical 1930s (*n* = 3). Predicted prophage regions were removed, and a maximum likelihood phylogenetic tree was constructed from 42,258 core SNPs. Nine STs were identified but the main lineage was ST624 with and minor (single base change in *recP*) ST variant ST837. We identified variants of P-*nga-ifs-slo* (P) associated with one of three combinations of key promoter residues, including the high-activity-associated A_−27_G_−22_T_−18_ (P; black). For *emm*76 (A) and *emm*77 (B), mutations were detected in *hasA* predicted to truncate HasA (H; black). (C) All *emm*81 isolates were predicted to express full-length HasA, but the ST624/ST837 lineage carried a mutation within *hasB* leading to a truncated HasB (H; gray). Branches are colored based on bootstrap support (scale bar provided). Scale bars represent substitutions per site. Isolates used as references for mapping indicated with black circles. Branches for lineages outside main lineages were shortened for presentation purposes (indicated by line breaks). C; collection, P; promoter key residue combination, H; full-length or truncated HasA or HasB.

The phylogeny of all available genome data for *emm*76, *emm*77, and *emm*81 confirmed the presence of diverse lineages associated with different MLSTs (Fig. 8A to C). In all genotypes, however, there was a dominant MLST lineage representing the majority of isolates: ST50 *emm*76, ST63 *emm*77, and ST624 *emm*81. Within the dominant MLST lineages of *emm*76 and *emm*77, there were sublineages that were associated with different P-*nga-ifs-slo* variants as well as loss of functional HasA through mutation.

We identified five different MLSTs within *emm*76 (Fig. 8A), but the majority of isolates (30/38) belonged to ST50, including both BSAC isolates. Recombination analysis of the ST50 lineage identified a sublineage that differed from other ST50 isolates by 19 regions of recombination (see Fig. S3). One of these regions encompassed P-*nga-ifs-slo*, conferring a P-*nga-ifs-slo* variant closely related to that of modern *emm*1 and *emm*89 with an identical high-activity promoter (subtype 3.1). This sublineage was dominated by PHE-2014/15 isolates and also contained the more recent of the two BSAC isolates (2008). All isolates in this sublineage, except one, also had a nonsense mutation within *hasA* of a C-to-T change at 646 bp, resulting in a premature stop codon after 215 aa, likely to render the isolates acapsular. Only one ST50 isolate outside this sublineage had the same *hasA* C646T change. All other *emm*76 isolates would express full-length HasA.

10.1128/mBio.02521-19.3 FIG S3 Recombination within ST50 *emm*76. All sequence data for *emm*76 (*n* = 38) was mapped the *de novo* assembled sequence of BSAC_bs448 (bold). The majority of isolates were ST50 (ST; white) and within this ST were two sublineages; the lower sublineage was associated the high-activity promoter A_−27_G_−22_T_−18_ (P; black) and truncated HasA (HasA/B; black). Gubbins analysis (boxed region on right) of ST50 isolates identified 19 regions of recombination across the genome in all isolates (red vertical lines) belonging to the lower sublineage compared to the top sublineage. One of these regions (highlighted grey) surrounded the P-*nga-ifs-slo* locus conferring the high-activity-associated promoter (P; black) with residues A_−27_G_−22_T_−18_ to the lower sublineage compared to low-activity A_−27_T_−22_C_−18_ (P; grey) in the top sublineage. The presence (black) or absence (white) of mobile prophage-associated superantigens (*speA*, -*C*, -*H*, -*I*, -*K*, -*L*, -*M*, and *ssa*) and DNAses (*sda*, *sdn*, *spd1*, *spd3*, *spd3v6*, and *spd4*) as well as antimicrobial resistance genes and mutant variants of regulators CovR, CovS, and RocA was also determined for each isolate. All isolates within the lower ST50 sublineage carried a variant of the prophage-associated DNAse *spd3* (*spd3v6*) (45) that is more divergent than other *spd3* variants, including the *spd3* variant carried by isolates belonging to the top ST50 sublineage. All lower sublineage isolates also carried the resistance gene *ermB* which was absent in other lineages, but they did not carry other antimicrobial elements found in the upper sublineage isolates. Sporadic truncated mutant variants of regulators CovR, CovS, and RocA (black) were also detected across the tree but were not associated with any specific lineages. Scale bar represents substitutions per site. Scale on boxed region represents position across the assembled BSAC_bs448 genome. Bootstrap values provided on major branches. Download FIG S3, TIF file, 1.8 MB. Copyright © 2019 Turner et al. 2019 Turner et al. This content is distributed under the terms of the Creative Commons Attribution 4.0 International license.

Two sublineages were also identified within the dominant *emm*77 lineage ST63 (Fig. 8B), and one was associated with the high-activity cluster P-*nga-ifs-slo* variant compared to predicted low-activity variants found in the other *emm*77 lineages. Recombination analysis predicted only two regions of recombination distinguishing the two sublineages: a region of 17,954 bp surrounding P-*nga-ifs-slo*, and a 173-bp region within a hypothetical gene (SPYH293_00394) (see Fig. S4). While all BSAC *emm*77 isolates (years 2001 to 2009) were ST63 with low-activity P-*nga-ifs-slo*, PHE isolates from 2014 to 2015 were almost evenly divided between the two sublineages, indicating a potential recent change in England/Wales. All ST63 isolates except two had a deletion of a T residue within a septa-poly(T) tract at 458 bp in *hasA*, predicted to truncate the HasA protein after 154 aa. The two exceptions were predicted to encode full-length HasA and were associated with low-activity P-*nga-ifs-slo* promoter variants. Although also not associated with high-activity P-*nga-ifs-slo* promoter variants, other lineages of *emm*77 also carried mutations within *hasA* that would truncate HasA; ST399 isolates carried an insertion of a T residue at 71 bp of the *hasA* gene resulting in a premature stop codon after 46 aa, and two ST133 isolates carried a G894A substitution resulting in a premature stop codon after amino acid residue 297.

10.1128/mBio.02521-19.4 FIG S4 Recombination within ST63 *emm*77. All sequence data for *emm*77 (*n* = 82) were mapped to the *de novo* assembled sequence of BSAC_bs150 (bold). The majority of isolates were ST63 (ST; white), or one single locus variant ST1125, and within this ST were two sublineages: the upper lineage associated with the high-activity promoter A_−27_G_−22_T_−18_ (P; black) and truncated HasA (H; black). Gubbins analysis (boxed region) of ST63 isolates identified two regions of recombination across the genome of all isolates (red vertical lines) belonging to the upper sublineage compared to the lower sublineage. One of these regions (highlighted grey) surrounded the P-*nga-ifs-slo* locus conferring the high-activity-associated promoter with residues A_−27_G_−22_T_−18_ (P; black) to the upper sublineage compared to low-activity G_−27_T_−22_T_−18_ (P; white) in the lower sublineage. The presence (black) or absence (white) of mobile prophage-associated superantigens (*speA*, -*C*, -*H*, -*I*, -*K*, -*L*, -*M*, and *ssa*) and DNAses (*sda*, *sdn*, *spd1*, *spd3*, *spd3v6*, and *spd4*) as well as antimicrobial resistance genes and truncated mutant variants of regulators CovR, CovS, and RocA was also determined for each isolate. The prophage-associated DNase *spd*3 was common to all upper sublineage ST63 isolates, and all except one of this sublineage carried the antimicrobial resistance gene *ermTR*. Sporadic truncated mutant variants of CovR, CovS, and RocA (black) were detected across the tree but were not associated with any specific lineages. Scale bar represents substitutions per site. Scale on boxed region represents position in the BSAC_bs150 assembly. Bootstrap values provided on main branches. Download FIG S4, TIF file, 2.5 MB. Copyright © 2019 Turner et al. 2019 Turner et al. This content is distributed under the terms of the Creative Commons Attribution 4.0 International license.

The *emm*81 population (*n* = 68) was more diverse with nine different sequence types (Fig. 8C), but the majority of isolates (41/68) were ST624 or the single locus variant ST837 (9/68; one SNP in *recP* allele) within the same lineage. ST171 was restricted to three historical isolates originally collected in 1938 and 1939. We did not detect any *hasABC* variations that would disrupt translation in *emm*81 lineages except for the dominant group of ST624/ST837, where we identified an A residue insertion at 128 bp in *hasB* resulting in a frameshift and premature stop codon after 50 aa. All ST624/ST837 isolates carried the high-activity cluster P-*nga-ifs-slo* variant identical to that seen in *emm*3 compared to all other lineages associated with other low-activity P-*nga-ifs-slo* variants. Recombination analysis identified extensive recombination had occurred within *emm*81 leading to the different levels of diversity, but we identified one region of recombination that distinguished the ST624/ST837 lineage from the closely related ST909 and ST117 populations (see Fig. S5). This region surrounded the P-*nga-ifs-slo* locus, suggesting ST624/ST837 gained the high-activity cluster P*-nga-ifs-slo* variant through recombination, like other *emm* types, potentially from *emm*3. The emergence of the high-activity P-*nga-ifs-slo* variant and truncated HasB ST624/ST837 lineage may be recent in England/Wales, as all BSAC isolates obtained prior to 2009 were outside this lineage.

10.1128/mBio.02521-19.5 FIG S5 Recombination within *emm*81. All sequence data for *emm*81 (*n* = 68) were mapped to the *de novo* assembled sequence of BSAC_bs229 (bold). The majority of isolates were ST624, with high-activity promoter A_−27_G_−22_T_−18_ (P; black) and truncated HasB (HasB; black). Gubbins analysis (boxed region) of ST624 isolates and closely related ST1059, ST117, ST909, and ST837, compared to BSAC_bs229, identified patterns of recombination across the genome in all isolates (red vertical lines, or blue vertical lines if unique to a single isolate). One of these regions (highlighted grey) surrounded the P-*nga-ifs-slo* locus conferring the high-activity-associated promoter with residues A_−27_G_−22_T_−18_ to the ST624/ST837 population compared to low-activity G_−27_T_−22_T_−18_ in all other isolates. The presence (black) or absence (white) of mobile prophage-associated superantigens (*speA*, -*C*, -*H*, -*I*, -*K*, -*L*, -*M*, and *ssa*) and DNAses (*sda*, *sdn*, *spd1*, *spd3*, *spd3v6*, and *spd4*) as well as antimicrobial resistance genes and truncated mutant variants of regulators CovR, CovS, and RocA was also determined for each isolate. The majority of all isolates carried the prophage-associated *speH*. Antimicrobial resistance genes were rarely detected in any ST. Scale bar represents substitutions per site. Scale on boxed region represents position in the BSAC_bs229 assembly. Bootstrap values provided on main branches. Download FIG S5, TIF file, 2.4 MB. Copyright © 2019 Turner et al. 2019 Turner et al. This content is distributed under the terms of the Creative Commons Attribution 4.0 International license.

### High-activity cluster P-*nga-ifs-slo* variants gained by recombination in *emm*94 and *emm*108.

Within *emm*94, we identified a P-*nga-ifs-slo* identical to that found in *emm*1 with high-activity promoter variant subtype 3.1. Phylogenetic analysis of 51 *emm*94 isolates identified a dominant lineage among England/Wales isolates separate to the single US isolate and two England/Wales isolates (see Fig. S6A) that belonged to ST89. Gubbins analysis predicted 11 regions of recombination in all lineage-associated isolates compared to the three outlying isolates, including one (22,648 bp) that encompassed P-*nga-ifs-slo*, transferring a high-activity A_−27_G_−22_T_−18_ P-*nga-ifs-slo* variant. All *emm*94 isolates contained an indel within *hasB* compared to the reference (H293), losing 6 bp and gaining 13 bp between 127 and 133 bp. This variation causes a frameshift and would truncate the HasB protein after 45 aa.

10.1128/mBio.02521-19.6 FIG S6 Recombination in *emm*94 and *emm*108. (A) In the PHE-2014/2015 (red) *emm*94 population, the majority (*n* = 51) form a lineage separate from two PHE-2014/2015 isolates and the single ABCs-2015 (blue) isolate. Gubbins analysis predicted 11 regions of recombination (red lines) in all the lineage associated isolates compared to the three other isolates. One of these regions (highlighted in grey) encompassed the P-*nga-ifs-slo* region. (B) Isolates of *emm*108 from the ABCs-2015 (blue) collection were of a different MLST (ST14) than PHE-2014/15 (red) (ST1088). The *hasB* gene was absent in the genomes of both ABCs-2015 isolates, and one had undergone recombination surrounding the P-*nga-ifs-slo* locus (shaded grey), as predicted by Gubbins analysis (shown on the right). Blue lines, predicted recombination unique to a single genome. Sequence data were mapped to the reference strain H293, also used as an outgroup for SNP cluster analysis. Scale bar represents SNPs. Download FIG S6, TIF file, 0.9 MB. Copyright © 2019 Turner et al. 2019 Turner et al. This content is distributed under the terms of the Creative Commons Attribution 4.0 International license.

We identified a similar high-activity cluster P*-nga-ifs-slo* variant within a single *emm*108 genome originating from the United States. Within the 9 isolates from PHE-2014/15 (*n* = 7) and ABCs-2015 (*n* = 2), there were two sequence types, ST1088 and ST14. ST14 was represented by the only two ABCs-2015 isolates, and we identified that both had lost the entire *hasB* gene, although *hasA* and *hasC* were still present (Fig. S6B). Additionally, one of the ABCs-2015 isolates had undergone recombination of a single ∼29,683-bp region surrounding the P-*nga-ifs-slo*, replacing P-*nga-ifs-slo* for one identical to that found in *emm*3 with high-activity promoter variant A_−27_G_−22_T_−18_ subtype 3.1.

### Mobile genetic elements and antimicrobial resistance.

The acquisition of mobile genetic elements such as prophages and transposons may also be influenced by capsule expression and can also influence the expansion and success of new lineages. We therefore determined the presence of prophage-associated superantigen and DNase genes as well as antimicrobial resistance genes to estimate the number of mobile genetic elements present within each isolate of the genotypes *emm*28, *emm*75, -76, -77, -81, -87, -94, and -108 (Fig. S3 to S5; Data Set S3). On average, there were 4.4 elements present in isolates predicted to express full-length HasABC compared to 2.5 elements present in isolates with *hasABC* gene mutations or gene absence, suggesting that the presence of capsule does not hinder mobile genetic elements. We also detected no link between lineages within these genotypes that had undergone P-*nga-ifs-slo* recombination and mobile factors, except within *emm*76 and *emm*77. Isolates belonging to the *emm*76 ST50 sublineage associated with HasA mutation and P-*nga-ifs-slo* recombination all carried the prophage-associate superantigen genes *speH* and *speI* as well as a diverse variant of the DNase *spd3* and the erythromycin resistance gene *ermB* (Fig. S3). This differed from the other ST50 isolates that carried another variant of *spd3* and multiple different resistance genes. The sublineage of ST63 *emm*77 associated with P-*nga-ifs-slo* recombination also carried *spd3*, and all, except one isolate, carried the erythromycin resistance gene *ermTR*; both genes were not common in other ST63 *emm*77 isolates (Fig. S4).

10.1128/mBio.02521-19.10 DATA SET S3 Details of *emm*28, *emm*75, *emm*76, *emm*77*, emm*81, *emm*87, *emm*94, and *emm*108 isolates used in this study. Download Data Set S3, XLSX file, 0.2 MB. Copyright © 2019 Turner et al. 2019 Turner et al. This content is distributed under the terms of the Creative Commons Attribution 4.0 International license.

## DISCUSSION

The emergence of new internationally successful lineages of S. pyogenes can be driven by recombination-related genome remodeling, as demonstrated by *emm*1 and *emm*89. The transfer of a P-*nga-ifs-slo* region conferring increased expression to the new variant was common to both genotypes. In the case of *emm*89, five other regions of recombination were identified in the emergent variant, one resulting in the loss of the hyaluronic acid capsule. Although, potentially, all six regions of recombination combined underpinned the success of the emergent *emm*89, we have shown here that recombination of P-*nga-ifs-slo* has occurred in other leading *emm* types as well as a high frequency of capsule loss through mutation. These data point to an association between genetic change affecting capsule and recombination affecting the P-*nga-ifs-slo* locus, conferring increased production of *nga-ifs-slo*; in some cases (notably *emm*87, *emm*89, and *emm*94), this has further been associated with an apparent fitness advantage and expansion within the population.

A number of genotypes were found to be associated with multiple variants of P-*nga-ifs-slo*. The majority of genotypes had P-*nga-ifs-slo* variants with the low-activity-promoter associated with three key residue variants: G_−27_T_−22_T_−18_ or A_−27_T_−22_C_−18_. Only *emm*1, *emm*3, and *emm*12 were exclusively associated with the high-activity A_−27_G_−22_T_−18_ variant. We have shown that the same high-activity promoter variant is present in isolates belonging to twelve other *emm* types, notably, *emm*76, *emm*77, *emm*81, *emm*87, and *emm*94, although this is not a consistent feature in these genotypes due to *emm* switching or recombination. We identified four combinations of the three key promoter residues and several subtypes of the 67-bp promoter that varied in bases other than those at the −27, −22, and −18 key positions. Although some subtypes were restricted to single genotypes, variation in the −40 base led to the subtype 2.2 of G_−27_T_−22_T_−18_ and subtype 3.2 of A_−27_G_−22_T_−18_. We measured the activity of NADase in representative strains and genotypes of these promoter variants and found that variation in the −40 base did not impact the activity conferred by the −27, −22, and −18 bases. Although we predicted the level of *nga* and *slo* expression based on the promoter variant, this may not relate to actual expression given the level of other genetic variation between genotypes. However, our consistent findings of lineages emerging following acquisition of the high-activity promoter variant supports the hypothesis that this confers some benefit that may relate to increased toxin expression.

Intriguingly, where we identified an acquisition of the high-activity promoter variant through recombination, these genotypes also had a genetic change in the capsule locus, likely rendering the organism unable to make capsule (*hasA* mutation) or only low levels of capsule (*hasB* mutation). To date, only *emm*4, *emm*22, and the emergent *emm*89 lineages are known to lack all three genes required to synthesize capsule. Here, we identified mutations that would truncate HasA and HasB in 35% of all isolates and 65% (35/54) of all genotypes. As the majority of isolates included in this study were invasive or sterile-site isolates, the findings further challenge the dogma that the hyaluronan capsule is required for full virulence of S. pyogenes and, in addition, lend credence to the possibility that the increased expression of NADase and SLO may in some way compensate for the lack of capsule (22). While capsule has been shown to underpin resistance to opsonophagocytic killing in the most constitutively hyperencapsulated genotypes such as *emm*18 (23, 24), there is less evidence that it contributes measurably to opsonophagocytosis killing resistance in other genotypes (3). Whether the loss of capsule synthesis is of benefit to S. pyogenes is uncertain; the capsule may shield several key adhesins used for interaction with host epithelium and fomites but may also act as a barrier to transformation with DNA. An accumulation of *hasABC* inactivating mutations has been identified during long-term carriage (25); although for some genotypes, capsule loss reduced survival in whole human blood, a high number of acapsular *hasA* mutants have also recently been found to be causing a high level of disease in children, including *emm*1, *emm*3, and *emm*12 (26).

The process of recombination in S. pyogenes is not well understood, and natural competence has only been demonstrated once and under conditions of biofilm or nasopharyngeal infection (27). We do not know if the six regions of recombination that led to the emergence of the new ST101 *emm*89 variant occurred simultaneously, although no intermediate isolates have been identified. The loss of the hyaluronic acid capsule in the new emergent *emm*89, along with our consistent findings of inactivating mutations associated with P-*nga-ifs-slo* transfer, indicates either (i) the process of recombination requires the inactivation of capsule, (ii) capsule-negative S. pyogenes requires high expression of *nga-ifs-slo* for survival, or (iii) that a capsule-negative phenotype combined with high expression of *nga-ifs-slo* provides a greater selective advantage to S. pyogenes.

The phylogeny of *emm*28, *emm*87, *emm*77, *emm*94, and *emm*108 indicated that mutations in *hasA* or *hasB* occurred prior to recombination of P-*nga-ifs-slo*, supporting the first hypothesis that prior capsule inactivation is required for recombination. There is no evidence, however, to suggest this was required for recombination in the *emm*1 population. It could be hypothesized that capsule acts as a barrier to genetic exchange, but there has also been a positive genetic association of capsule to recombination rates (28). A positive association may, however, be related only to species expressing antigenic capsule, whereby recombination is required to introduce variation for immune escape.

The *hasC* gene is not essential for capsule synthesis (29), because a paralog of *hasC* exists within the S. pyogenes genome. A paralog for *hasB* (*hasB.2*) also exists elsewhere in the S. pyogenes chromosome and can act in the absence of *hasB* to produce low levels of capsule (30), but *hasA* is absolutely essential for capsule synthesis (29). The mutations in *hasA* in *emm*28 and *emm*87 have been previously noted and confirmed to render the isolates acapsular (26, 31). Not all acapsular isolates were found to carry the high-activity promoter of *nga-ifs-slo*, despite being invasive, perhaps refuting the hypothesis that the high-activity *nga*-*ifs-slo* promoter is essential for the survival of acapsular S. pyogenes. High expression of *nga-ifs-slo* may also occur through other mechanisms, for example, through mutation in regulatory systems. We looked at the sequences of *covRS* and *rocA*, known to negatively regulate *nga-ifs-slo*, in all isolates (see Data Set S2 in the supplemental material) and identified some *emm*-type specific variants, consistent with our previous findings (11). We did not identify any other genotypes where all isolates carried truncation mutations in *rocA*, such as *emm*3 and *emm*18 that were previously confirmed to affect function and increase expression of *rocA-covR*-regulated virulence factors (23, 32), consistent with other findings (14). It is unclear as to whether the amino acid changes in found in other genotypes would affect function of *rocA* as well as *covR* and *covS*, and this requires further work.

Interestingly, we identified that the capsule locus is also a target for recombination as, similarly to *emm*89, isolates within *emm*28 and *emm*87 had undergone recombination of this locus and surrounding regions, varying in length and restoring capsule synthesis in *emm*28. Isolated examples of *hasA* or *hasB* gene loss were identified in some genotypes, such as *emm*108, possibly due to internal recombination and deletion.

Only two *emm*4 isolates and one *emm*22 isolate were found to have P-*nga-ifs-slo* variants that were not A_−27_T_−22_G_−18_ high-activity promoter variants, and interestingly, these isolates carried the *hasABC* genes, typically absent in *emm*4 and *emm*22. The high genetic distance of these isolates to other *emm*4 and *emm*22 genomes indicated potential *emm* switching of the *emm*4 or *emm*22 genes into different genetic backgrounds. The single *emm*28 with a high-activity P-*nga-ifs-slo* variant may also be an example of this, and was one of four *emm*28 isolates that did not cluster with the two main *emm*28 lineages. Although we excluded them from our analysis, as we focused on recombination within the two main lineages, the presence of highly diverse variants within genotypes and the potential for *emm*-switching warrants further investigation, particularly as the most promising current vaccine is multivalent toward common M types (33).

All other genotypes carrying the high-activity P-*nga-ifs-slo* variant were found to have undergone recombination of this region: *emm*28, *emm*75, *emm*76, *emm*77, *emm*81, *emm*87, *emm*94, and *emm*108, as well as the previously described *emm*1 and *emm*89.

Within *emm*87, we identified three isolates outside the main population lineage that represented the oldest isolates in the collection: two from 2001 (different geographical locations within England) and one NCTC strain from ∼1970 to 1980 (NCTC12065). A single region of recombination surrounding the P-*nga-ifs-slo* locus distinguished the main population lineage from the three older isolates, consistent with a recombination event, but due to a lack of earlier isolates of *emm*87, we could not confirm a recombination-related shift in the population, as reported previously for *emm*89 and *emm*1.

The existence of two lineages within the contemporary *emm*28 suggests that one has not yet displaced the other, although the MEW123-like lineage was predominantly US isolates, consistent with recent findings (15). The P-*nga-ifs-slo* region with the high-activity-associated A_−27_T_−22_T_−18_ and acquired through recombination by the MEW123-like lineage was identical to that found in *emm*78, indicating *emm*78 as the potential genetic donor. We found *emm*78 to have high levels of NADase activity, as predicted, and interestingly, similarly to *emm*28, all eight *emm*78 isolates were acapsular due to a deletion within the *hasABC* promoter region extending into *hasA*. This again may support the hypothesis that capsule-negative S. pyogenes requires high expression of *nga-ifs-slo* for survival.

A strength of this study was the systematic longitudinal sampling over a 10-year period; as expected, this again identified the shift in the *emm*89 population. Other *emm* types exhibited lineages with different P-*nga-ifs-slo* variants, and those with the more active promoter variant did appear to become dominant over time, similarly to *emm*1 and the emergent *emm*89 lineages. For example, the high-activity P-*nga-ifs-slo* ST63 lineage of *emm*77 was not detected in England/Wales isolates prior to 2014 and 2015. Similarly, the high-activity P-*nga-ifs-slo* variant *emm*81 ST646/ST837 lineage was represented by only a single isolate (of six) collected between 2001 and 2009 but became dominant by 2014 to 2015 in England/Wales and the United States. *emm*75 was the 6th most common genotype in England/Wales in 2014 to 2015 and dominated by high-activity P-*nga-ifs-slo* variant ST150 lineage yet was less common in the United States, where ST49 with low-activity P-*nga-ifs-slo* is dominant. A high prevalence of *emm*94 was also found in England/Wales between 2014 and 2015 but was rare in the United States (only 1 isolate). Our analysis of this genotype indicated there has been a recombination-related change in the population, as we detected 11 regions of predicted recombination, including P-*nga-ifs-slo*, potentially conferring high toxin expression. The other ten regions of recombination may also provide advantages to this lineage along with a potential low level of capsule through *hasB* mutation.

Other factors may also contribute to the success of emergent new lineages, including mobile prophage-associated virulence factors and antimicrobial resistance genes. Acquisition of mobile genetic elements did not appear to be affected by capsule loss; indeed, fewer mobile genetic element-associated factors were detected in isolates with capsule gene mutations than in isolates with functional capsule genes. A number of bacteriophages that target S. pyogenes encode a hyaluronidase thought to allow the bacteriophage to access the bacterial surface by degrading the outer capsule layer (34); therefore, recombination of these elements is likely to be different from gene transfer of core genetic regions, such as P-*nga-ifs-slo*.

We did, however, identify an association in the lineages of *emm*76 and *emm*77 with prophage-associated virulence factors and antimicrobial resistance genes. It is possible that the superantigens *speH*, *speI*, and DNase *spd3* may also have contributed to the success of the lineages that had undergone P-*nga-ifs-slo* recombination. Of concern is that both *emm*76 and *emm*77 carried genes for resistance to tetracycline and erythromycin, which were rarer in other genotypes. If the acapsular/high-toxin-expressing lineages do expand in the population, it will be important to monitor the levels of antimicrobial resistance in these lineages. This is also true for *emm*108, as *tetM* was detected in all isolates, but the presence of antimicrobial resistance genes was rare in *emm*28, *emm*75, *emm*81, *emm*87, and *emm*94, regardless of lineage.

The development and boosting of circulating antibodies to SLO is often used as a diagnostic biomarker of recent S. pyogenes infection and is known to be more specific to throat rather than skin infections. The genomic analysis provides explanation for this historic and well-recognized association between anti-streptolysin O (ASO) titers and disease patterns, due to known tissue tropism of S. pyogenes *emm* types. Whether the alteration of SLO activity in different S. pyogenes strains might render such a test more or less specific will be of interest, although it may explain observed differences in ASO titers between genotypes (35). There is also the possibility that other beta hemolytic streptococci might acquire similarly active SLO production, reducing the specificity of ASO titer to S. pyogenes.

Our genomic analysis has uncovered convergent evolutionary pathways toward capsule loss and recombination-related remodeling of the P-*nga-ifs-slo* locus in leading contemporary genotypes. This suggests that a combination of capsule loss and gain of high *nga-ifs-slo* expression provide a greater selective advantage than either of these phenotypes alone. Acquisition of the high-activity promoter led to pandemic *emm*1 and *emm*89 clones that are dominant and highly successful. Active surveillance of the lineages comprising *emm*76, *emm*77, *emm*81, *emm*87, *emm*94, and *emm*108 is required to determine if capsule loss/reduction and recombination of P-*nga-ifs-slo* toward high expression will trigger expansion toward additional pandemic clones in the next few years.

## MATERIALS AND METHODS

### Isolates.

Three hundred forty-four isolates of S. pyogenes associated with bloodstream infections and submitted to the British Society for Antimicrobial Chemotherapy (BSAC; http://www.bsacsurv.org) from 11 different sites across England between 2001 and 2011 were subjected to whole-genome sequencing (see Data Set S1 in the supplemental material). All BSAC isolates were tested for antibiotic susceptibility using the BSAC agar dilution method to determine MICs (36).

A further six isolates were sequenced from a historical collection of S. pyogenes originally collected in the 1930s from puerperal sepsis patients at Queen Charlottes Hospital, London, UK; one *emm*28 isolate from 1938 (ERR485803), two *emm*75 isolates from 1937 (ERR485807) and 1939 (ERR485820), and three *emm*81 isolates from 1938 (ERR485805) and 1939 (ERR485801, ERR485802).

### Genome sequencing.

Streptococcal DNA was extracted using the QIAxtractor instrument according to the manufacturer’s instructions (Qiagen, Hilden, Germany) or manually using a phenol-chloroform method (37). DNA library preparation was conducted according to the Illumina protocol, and sequencing was performed on an Illumina HiSeq 2000 with 100-cycle paired-end runs.

Genomes were *de novo* assembled using Velvet with the pipeline and improvements found at https://github.com/sanger-pathogens/vr-codebase and https://github.com/sanger-pathogens/assembly_improvement (38). Annotation was performed using Prokka. *emm* genotypes were determined from the assemblies, and multilocus sequence types (MLSTs) were identified using the MLST database (https://pubmlst.org/spyogenes/) and an in-house script (https://github.com/sanger-pathogens/mlst_check). New MLSTs were submitted to the database (https://pubmlst.org/).

### Genome sequence analysis.

Sequence reads were mapped using SMALT (https://www.sanger.ac.uk/science/tools/smalt-0) to the completed *emm*89 reference genome H293 (HG316453.2) (3), as this genome contains no known prophage regions. Other reference genomes were also used where indicated with predicted prophage regions (Table S1) excluded to obtain “core” SNPs. Maximum likelihood phylogenetic trees were generated from aligned core SNPs using RAxML (39) with the GTR substitution model and 100 bootstraps. Regions of recombination were predicted using Gubbins analysis with the default parameters (40). Branches of phylogenetic trees were colored according to bootstrap support using iTOL (41).

10.1128/mBio.02521-19.7 TABLE S1 Reference genomes used for mapping in this study and excluded prophage regions. Download Table S1, DOCX file, 0.1 MB. Copyright © 2019 Turner et al. 2019 Turner et al. This content is distributed under the terms of the Creative Commons Attribution 4.0 International license.

Other genome sequence data were obtained from the short read archive. We combined data collected across England and Wales through Public Health England during 2014 and 2015 (PHE-2014/15) supplied by Kapatai et al. (13) and Chalker et al. (12) from invasive and noninvasive S. pyogenes isolates. We also used data supplied by Chochua et al. (14) collected by Active Bacterial Core Surveillance USA in 2015 (ABCs-2015) from invasive S. pyogenes isolates. ABCs-2015 sequence data were preprocessed by Trimmomatic (42) to remove adapters and low-quality sequences. PHE-2014/15 had already been preprocessed (12, 13). Genome data from these collections were assembled *de novo* using Velvet (assembly statistics provided in Data Set S2), and any isolates with greater than 2.2 Mbp total assembled length and/or more than 500 contig numbers were excluded. We also used data from Turner et al. (11) of invasive and noninvasive isolates from the Cambridgeshire region, UK, and collected through Cambridge University Hospital (CUH). We relied on the *emm* types determined during the original studies and excluded any data where the *emm* type was uncertain or negative. The genes *hasA*, *hasB*, *hasC*, *covR*, *covS*, and *rocA*, and the P-*nga-ifs-slo* were extracted from the assembled genome using *in silico* PCR (https://github.com/simonrharris/in_silico_pcr). Capsule locus and P-*nga-ifs-slo* variants were also confirmed through manual inspection of mapping data where genotype could not be accurately determined from the assembly.

Mapping of *emm*76, *emm*77, and *emm*81 sequence data was performed using *de novo* assembled genome data from one BSAC collection isolate representing the equivalent genotype. Prophage regions were predicted using PHASTER (43) and removed before SNP extraction.

Antimicrobial resistance genes were identified by srst2 (44) using the ARG-ANNOT database (ARGannot_r2.fasta) (45). The presence of prophage-associated superantigen genes *speA*, *speC*, *speH*, *speI*, *speL*, *speM*, and *ssa* was determined using srst2 and the feature database previously used by Chochua et al. (14) available at https://github.com/BenJamesMetcalf. The presence of prophage-associated DNAses genes *sda*, *sdn*, *spd1*, *spd3*, *spd3v6*, and *spd4* was also determined using srst2 by adding regions of these genes to the feature database. Representative alleles of these DNase genes were taken from previous analysis (46) to identify regions that would detect all variants of each DNase, except we included *spd3v6* separate from *spd3* as it represents a divergent allele to *spd3*. Sequences used are available at Mendeley (https://data.mendeley.com/datasets/hzwjkj2gtp/1).

### NADase activity.

Activity of NADase was measured in culture supernatants as previously described (3). Activity was determined as the highest dilution capable of hydrolyzing NAD^+^. Isolates were selected from the BSAC collection to represent different promoter variants for which there were three or more isolates available that were lacking mutations in regulatory genes.

### Data availability.

Sequence data have been submitted to the European Nucleotide Archive (ENA) (www.ebi.ac.uk/ena) as accession numbers ERS361826 to ERS379364, ERR1359331 to ERR485881, ERS361826 to ERS379364, and SRR5853328 to SRR5858742 (listed in Data Sets S1 and S2 in the supplemental material).
