# Supplementary figures and images for: The Emergence of Successful Streptococcus pyogenes Lineages through Convergent Pathways of Capsule Loss and Recombination Directing High Toxin Expression
Source: mBio. 2019 Dec 10;10(6):e02521-19. doi: 10.1128/mBio.02521-19 (PMC6904876; doi:10.1128/mBio.02521-19)

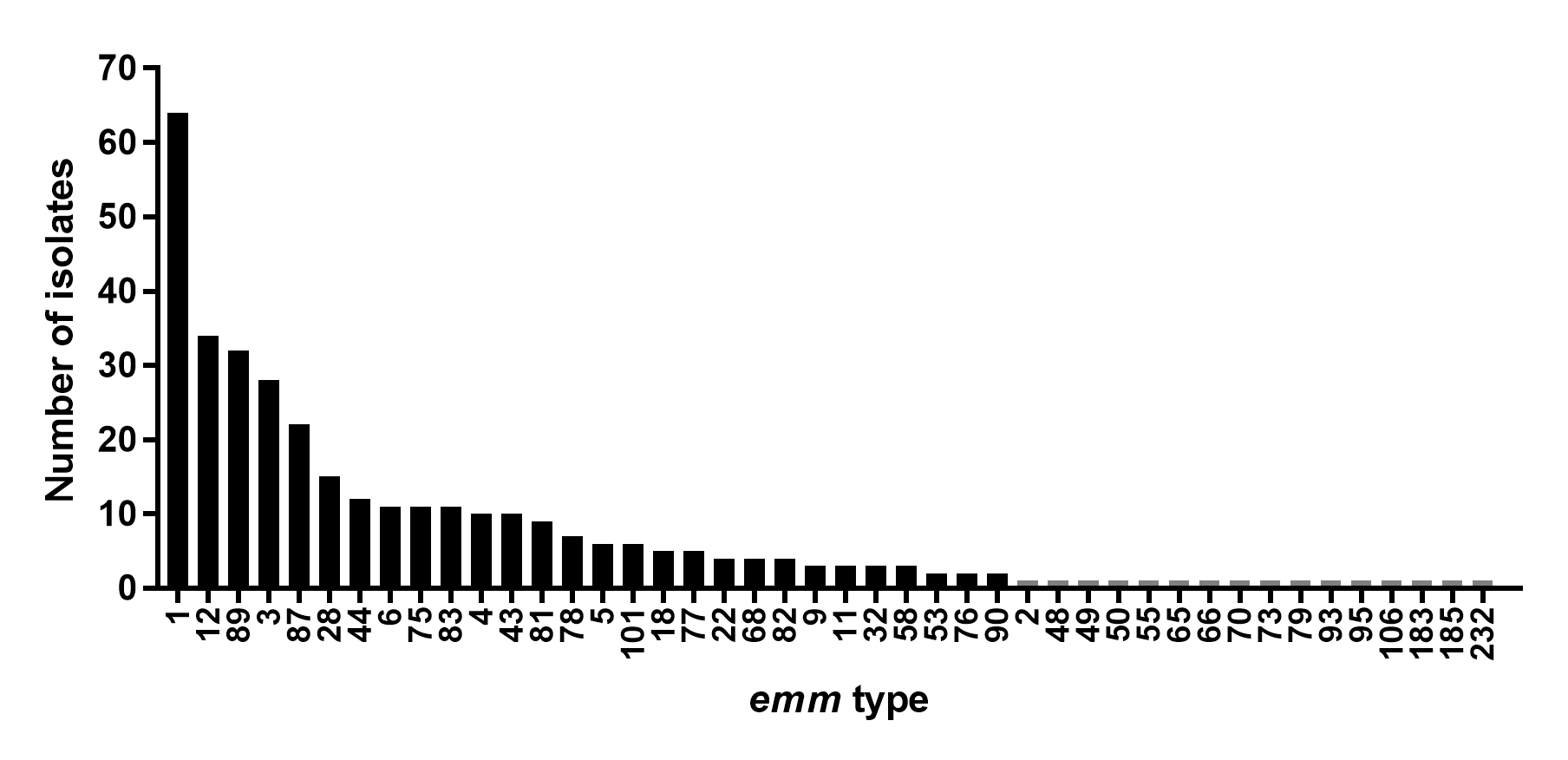

Supplement: FIG S1 [file mBio.02521-19-sf001.tif]

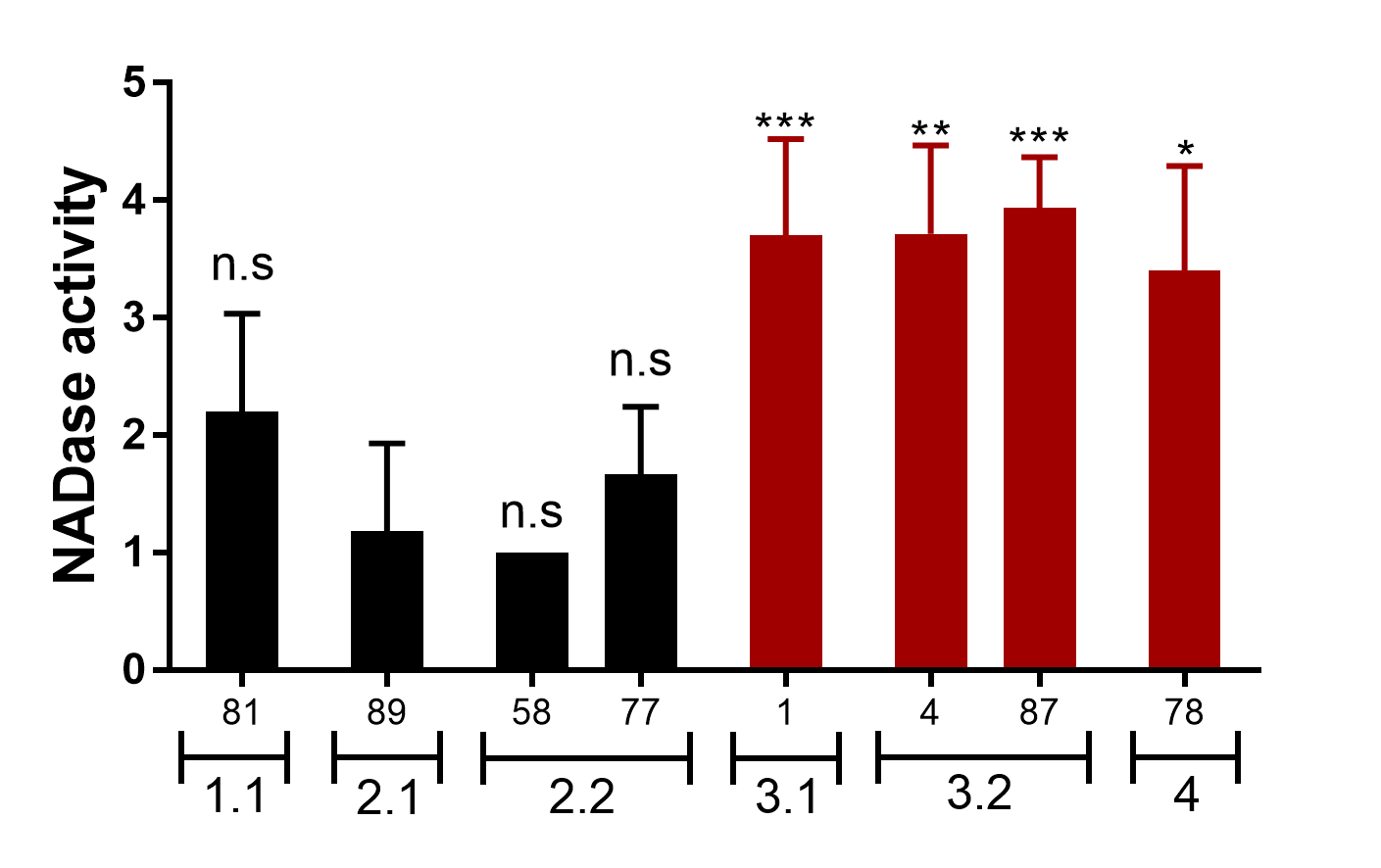

Supplement: FIG S2 [file mBio.02521-19-sf002.tif]

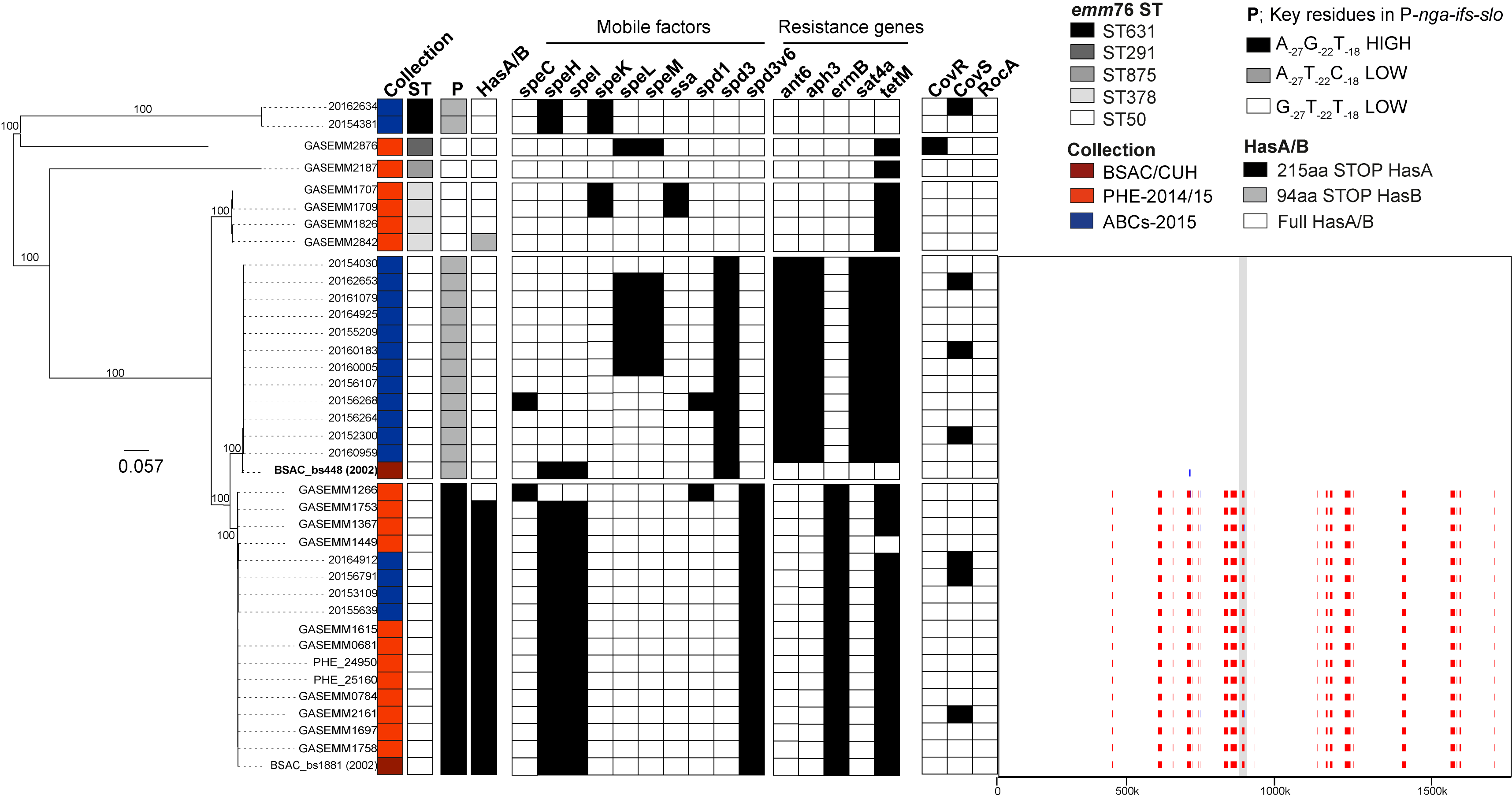

Supplement: FIG S3 [file mBio.02521-19-sf003.tif]

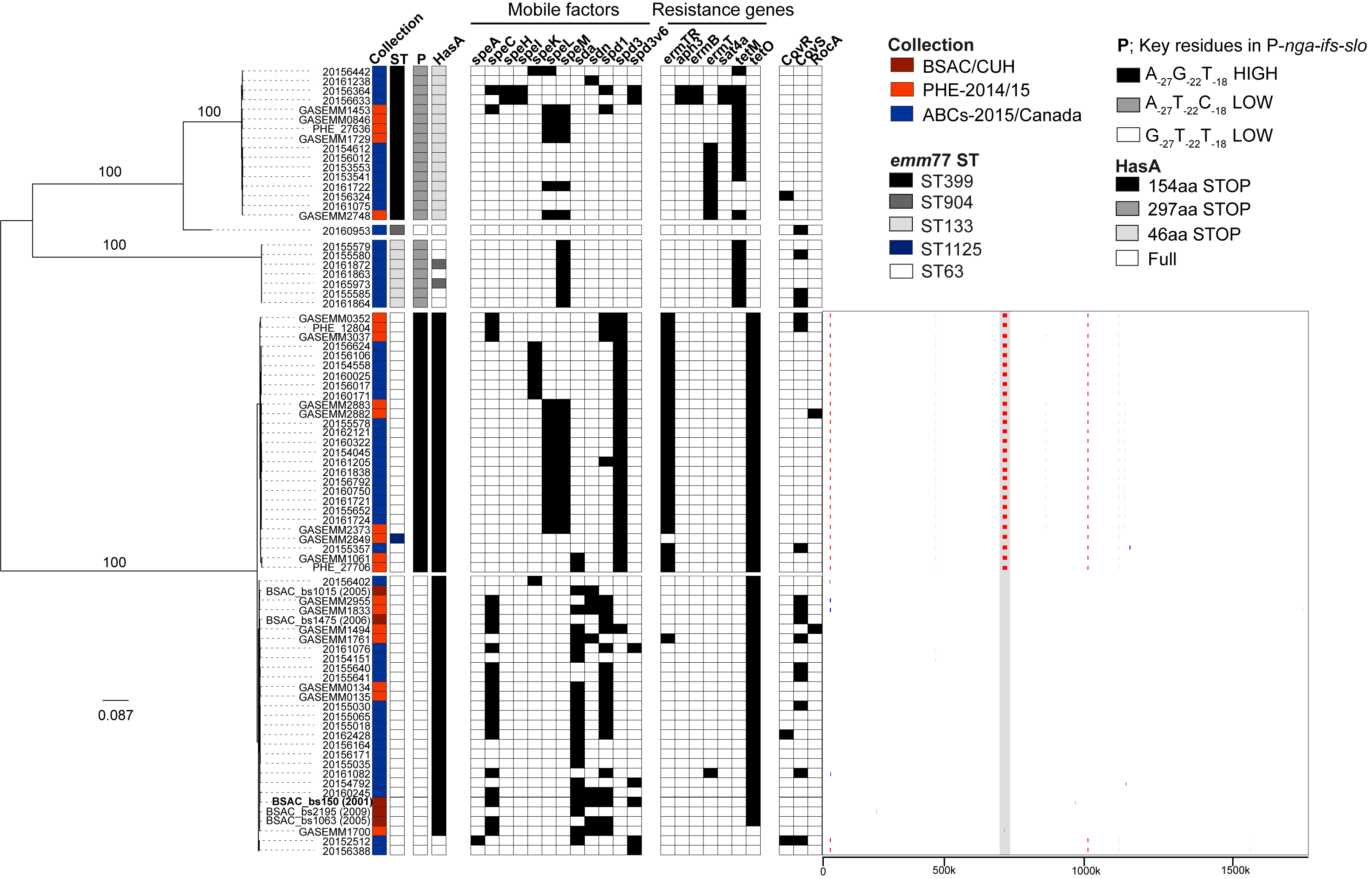

Supplement: FIG S4 [file mBio.02521-19-sf004.tif]

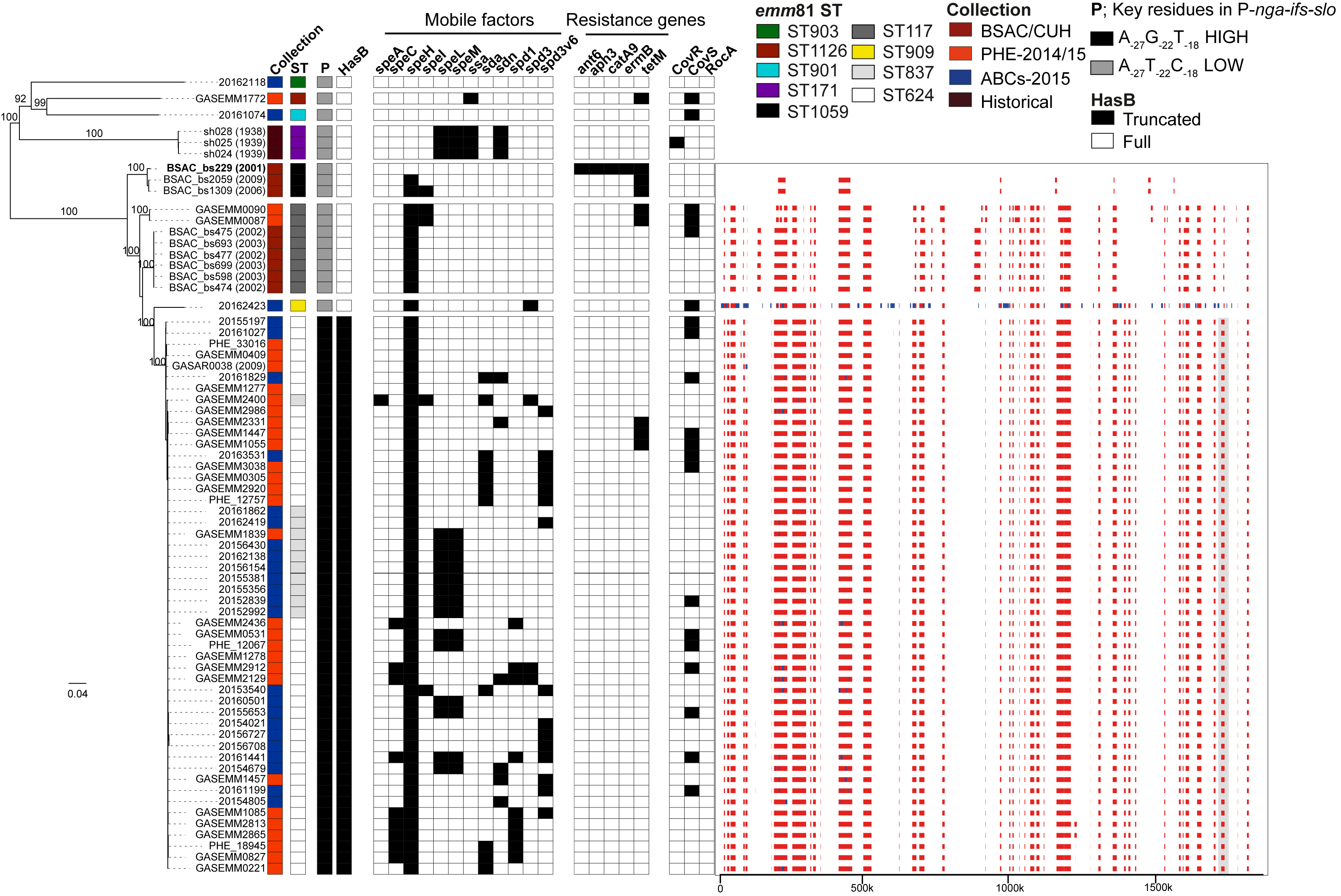

Supplement: FIG S5 [file mBio.02521-19-sf005.tif]

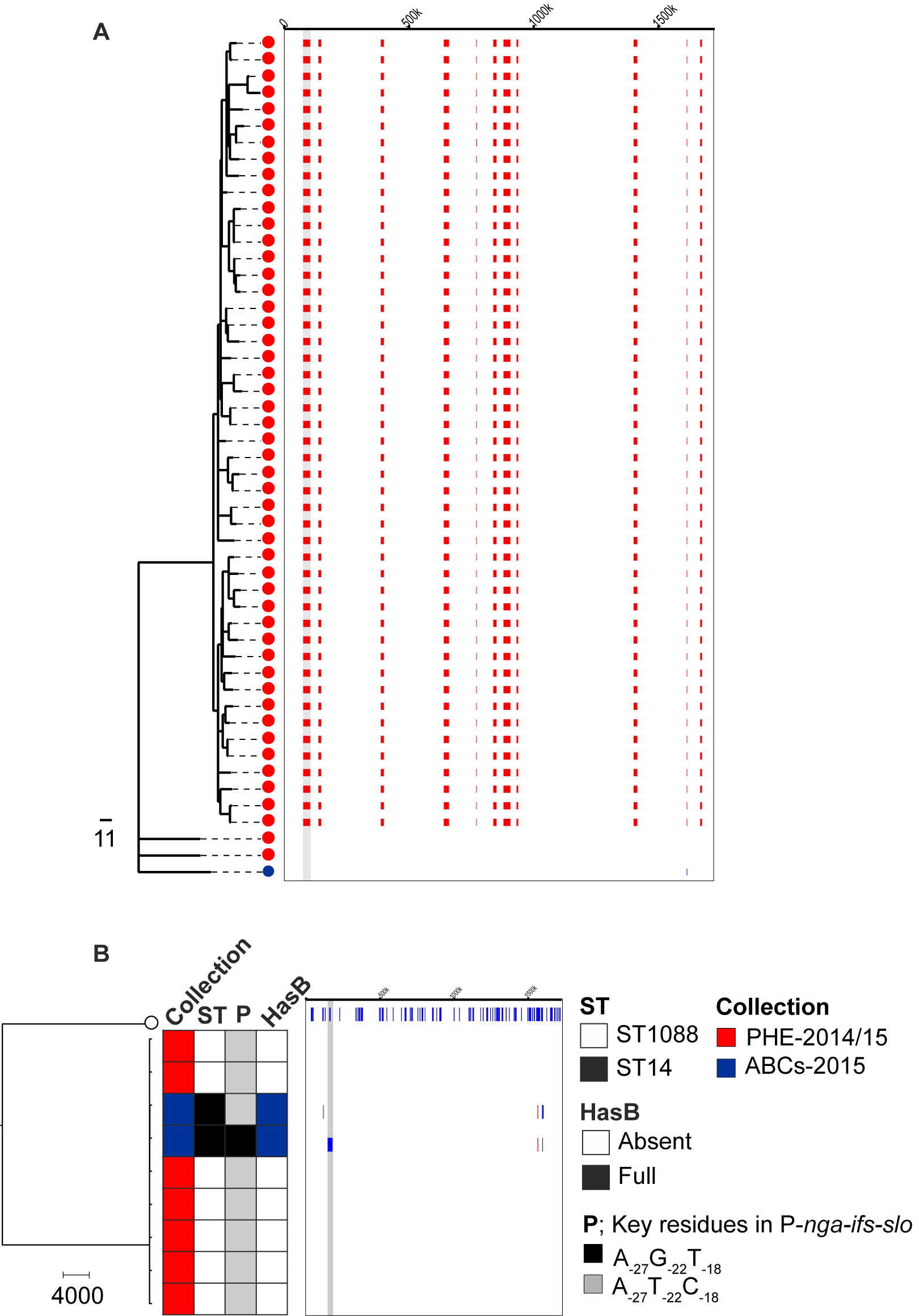

Supplement: FIG S6 [file mBio.02521-19-sf006.tif]
