# Supplementary material for: The Emergence of Successful Streptococcus pyogenes Lineages through Convergent Pathways of Capsule Loss and Recombination Directing High Toxin Expression
Source: mBio. 2019 Dec 10;10(6):e02521-19. doi: 10.1128/mBio.02521-19 (PMC6904876; doi:10.1128/mBio.02521-19)
Supplement: TABLE S1 [file mBio.02521-19-st001.docx]

**Supplementary Table 1. Reference genomes used for mapping to in this study and excluded prophage regions**

| **Reference strain** | | ***emm* type** | **Prophage locations** |
| --- | --- | --- | --- |
| H293 (HG316453.2) | 89 | | None |
| MGAS6180 (CP000056) | 28 | | 986212-1032479 |
|  |  | | 1226909-1269223 |
|  |  | | 1807177-1821524 |
|  |  | | 1845845-1857621 |
|  |  | | 1081040-1092149 |
|  |  | | 1286346-1322672 |
| STAB090229 (CP020027) | 75 | | 723790-761364 |
|  |  | | 1138154-1180947 |
|  |  | | 1473588-1512859 |
| NGAS743  (CP007560.1) | 87 | | 547772-585345 |
|  |  | | 709624-756401 |
|  |  | | 1199244-1242181 |
|  |  | | 1253797-1293056 |
